# From NiMoO_4_ to γ-NiOOH: Detecting the Active Catalyst Phase by Time Resolved *in Situ* and *Operando* Raman Spectroscopy

**DOI:** 10.1021/acsnano.1c04126

**Published:** 2021-08-12

**Authors:** Robin N. Dürr, Pierfrancesco Maltoni, Haining Tian, Bruno Jousselme, Leif Hammarström, Tomas Edvinsson

**Affiliations:** † Department of Chemistry, Physical Chemistry, Ångström Laboratory, Uppsala University, Box 523, 751 20 Uppsala, Sweden; ‡ Department of Materials Science and Engineering, Solid State Physics, Ångström Laboratory, Uppsala University, Box 35, 751 03 Uppsala, Sweden; § Université Paris-Saclay, CEA, CNRS, NIMBE, LICSEN, 91191 Gif-sur-Yvette, France

**Keywords:** electrocatalysis, alkaline water splitting, nickel molybdate, molybdenum leaching, *in situ* catalyst formation, nanostructures, time-resolved *operando* Raman spectroscopy

## Abstract

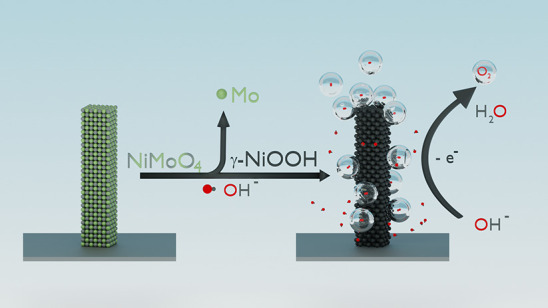

Water electrolysis powered by renewable energies is a promising technology to produce sustainable fossil free fuels. The development and evaluation of effective catalysts are here imperative; however, due to the inclusion of elements with different redox properties and reactivity, these materials undergo dynamical changes and phase transformations during the reaction conditions. NiMoO_4_ is currently investigated among other metal oxides as a promising noble metal free catalyst for the oxygen evolution reaction. Here we show that at applied bias, NiMoO_4_·H_2_O transforms into γ-NiOOH. Time resolved *operando* Raman spectroscopy is utilized to follow the potential dependent phase transformation and is collaborated with elemental analysis of the electrolyte, confirming that molybdenum leaches out from the as-synthesized NiMoO_4_·H_2_O. Molybdenum leaching increases the surface coverage of exposed nickel sites, and this in combination with the formation of γ-NiOOH enlarges the amount of active sites of the catalyst, leading to high current densities. Additionally, we discovered different NiMoO_4_ nanostructures, nanoflowers, and nanorods, for which the relative ratio can be influenced by the heating ramp during the synthesis. With selective molybdenum etching we were able to assign the varying X-ray diffraction (XRD) pattern as well as Raman vibrations unambiguously to the two nanostructures, which were revealed to exhibit different stabilities in alkaline media by time-resolved *in situ* and *operando* Raman spectroscopy. We advocate that a similar approach can beneficially be applied to many other catalysts, unveiling their structural integrity, characterize the dynamic surface reformulation, and resolve any ambiguities in interpretations of the active catalyst phase.

Climate change and anthropogenic global warming, caused by rising amount of greenhouse gases, are two of the most urgent challenges we are facing in the 21st century.^1,2^ A net addition of CO_2_ into the atmosphere, generated by burning fossil fuel, is a known contributor to the greenhouse effect. A transition to renewable fossil free or carbon neutral fuels is therefore highly desired and urgent.^3−6^ Rising effort is made to store renewable energy in chemical bonds where hydrogen, with its high gravimetric energy density, is a very promising energy carrier.^7−9^ Unfortunately, most of the hydrogen utilized today is produced by steam re-forming from methane, in which methane is transformed at high temperatures to hydrogen with an accompanying release of carbon dioxide.^4,10^ An interesting and carbon neutral method to generate hydrogen is sustainable water electrolysis, in which water is split into molecular oxygen and hydrogen with the use of renewable electricity.^11^ By powering of this process with solar or wind energy, the obtained hydrogen is carbon dioxide neutral and fully renewable, neglecting the carbon footprint of the manufactured wind, solar, and electrolysis devices.^4,12−14^ Not only is splitting water photochemically, electrochemically, or photoelectrochemically of high interest for hydrogen generation, but a similar approach can also be utilized for the reduction of carbon dioxide to different chemicals (carbon monoxide, methane, or smaller organic molecules) or the reduction of nitrogen to form ammonia in a carbon neutral process.^12,15−21^ The formation of hydrogen, carbon monoxide, methane, organic molecules, or ammonia from N_2_ is done via reduction of their starting products and is thus taking place at the cathode of the electrochemical cell. The reaction at the anode is instead oxidation of water or hydroxide ions to molecular dioxygen. In order to successfully establish sustainable water electrolysis, the more sluggish oxygen evolution by oxidation of water compared to, for example, the hydrogen evolution reaction (HER) at the cathode has to be accelerated.^22,23^ Conducting water splitting in more alkaline media can increase the catalyst’s maximum turnover frequency or decrease its Tafel slope for the oxygen evolution reaction (OER).^24−27^ Splitting water in alkaline media has another major advantage. In contrast to water splitting in acidic media, where efficient catalysts are limited to rare metal oxides like IrO_2_ or RuO_2_, alkaline media allows the use of efficient and stable catalysts from earth abundant transition metal compounds. Among others, NiMoP_2_, NiCo_2_O_4_, CoFe, CoTiO_2_, MoNi_4_, Ni_3_S_2_, Co(OH)_2_, Ni(OH)_2_, NiFe layered double hydroxide or metal molybdates are promising candidates.^23,28,37−40,29−36^

Due to the facile and versatile way of synthesizing nickel or cobalt molybdates in different nanostructures and hence different surface areas, this catalyst has recently been investigated closely as catalyst for OER and HER in alkaline media. Those compounds are versatile and can also only act as intermediate for the actual catalyst after an additional annealing and reduction step of the starting compound.^7,35,37,41^ Metal molybdates have already been synthesized on metal foams or as powders and investigated in a large number of publications.^7,30,45−52,35−38,41−44^ However, the stability of the metal molybdate or the role of molybdenum is not fully clarified.^35,36,38^ It is essential to know the active catalytic phase and the role of added elements. Since the catalytic performance of a material can in general be improved by either providing more active sites or improving the single active site, understanding the role of each element is fundamental for further enhancements.

## Results and Discussion

The stability of metal molybdates and the role of molybdenum are clearly not sufficiently investigated for the catalyst under operating conditions and form the motivation for the present study. A main question is whether molybdenum is still present at the activated catalyst surface or if its major role is that of a structural forming agent providing a highly porous and enlarged surface area of the catalyst. Furthermore, it is important to investigate how variations in synthesis condition are affecting the catalyst properties and also, here, how this effects the reported stability and function of the molybdate. The synthesis temperature as well as the holding time is in most publications reported, while less information is given on the heating rate that is known to have a strong influence on the formed materials. In order to investigate a catalyst, which can be used in a commercial electrolyzer cell free of scarce metals, nickel was chosen as the metal for the metal molybdate. In contrast to cobalt, nickel is not listed as critical raw material in the 2017 report of the European Union.^53^ In this work we perform a detailed study on the stability and role of molybdenum in nickel molybdate hydrate grown on nickel foam for use as oxygen evolution catalyst in alkaline media together with the influence of the heating rate during the synthesis. The role of molybdenum and the activated form of NiMoO_4_ grown on open porous nickel foam is investigated *ex situ* before and after catalysis with various analytical tools, as well as with time-resolved *in situ* and *operando* Raman spectroscopy during catalysis. On the basis of the heating ramp during the synthesis, the samples are denoted as NiMoO_4_@Nif-0.5 (0.5 °C min^–1^), NiMoO_4_@Nif-2 (2 °C min^–1^), and NiMoO_4_@Nif-5 (5 °C min^–1^).

### Characterization of the As-Prepared Samples

**Figure 1:**
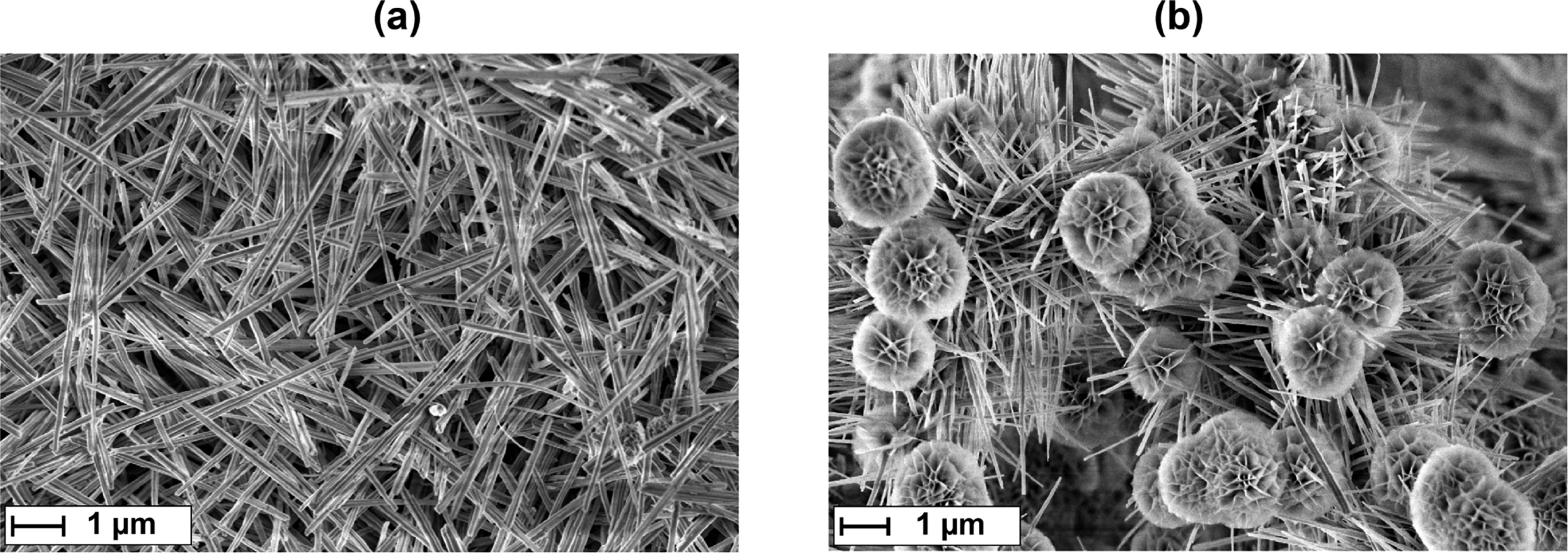
Secondary electron imaging of the as-synthesized NiMoO_4_@Nif, showing different nanostructures. Both images were taken at similar magnifications (17000–20000×) and accelerating voltage (2–3 kV). (a) NiMoO_4_@Nif-2, exhibiting exclusively nanorod shaped NiMoO_4_. (b) NiMoO_4_@Nif-5 showing both nanorod and nanoflower shaped NiMoO_4_.

Scanning electron microscopy (SEM) revealed a uniform coated nickel foam with nanostructures of the as-synthesized NiMoO_4_·H_2_O. The secondary electron images show two different nanostructures. Nanorod shaped structures with a diameter of approximately 80–170 μm, which were present on all samples and nanoflower shaped structures, build up from nanoflakes, which were not observed on NiMoO_4_@Nif-2, but with rising ratio for NiMoO_4_@Nif-0.5 and NiMoO_4_@Nif-5 (Figure 1, SI Figure 1, SI Figure 4, SI Figure 7). These results show a clear influence of the heating ramp on the as-synthesized material. On the basis of the shape of their nanostructures, we denote them as rod-NiMoO_4_ and flower-NiMoO_4_. Energy dispersive X-ray spectroscopy (EDX) (SI Figure 2, SI Figure 5, SI Figure 8) shows homogeneous distribution of nickel, molybdenum, and oxygen in both the rod-NiMoO_4_ and flower-NiMoO_4_ for all samples. Interesting to note is that the atomic ratio of nickel to molybdenum is increased for flower-NiMoO_4_ (Ni:Mo ≥ 1.53, detected by EDX point ID) compared to rod-NiMoO_4_ (Ni:Mo ≤ 1.36, also detected by EDX point ID) (SI Figure 3, SI Figure 6, SI Figure 9) and listed in SI Table 1. The same trend is visible in the overall map sum spectra of the samples as well as with X-ray photoelectron spectroscopy (XPS), as shown below. This is already supporting a compositional difference between the rod and flower shaped NiMoO_4_.

**Figure 2:**
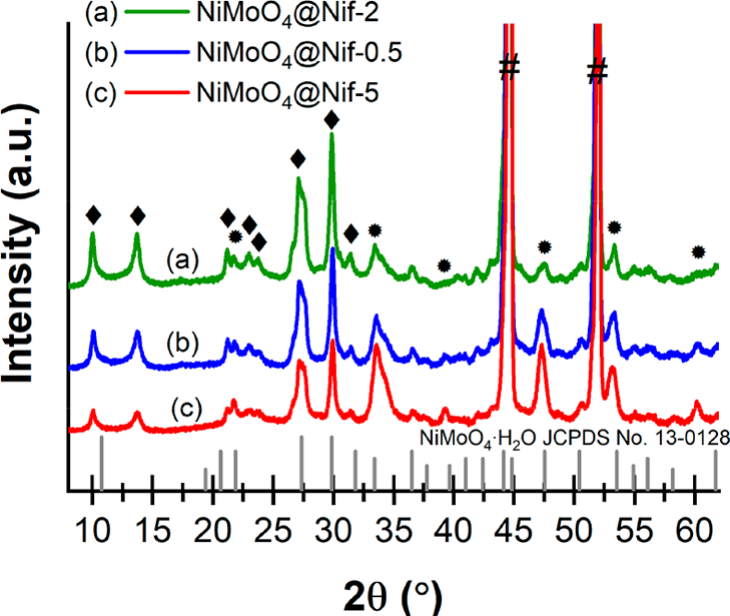
XRD patterns of the different NiMoO_4_@Nif samples for (a) NiMoO_4_@Nif-2, (b) NiMoO_4_@Nif-0.5, and (c) NiMoO_4_@Nif-5. Diffraction signals from the rod-NiMoO_4_ are indicated with “◆”, whereas flower-NiMoO_4_ signals are indicated with “●”. Signals from the nickel foam are highlighted with “#”. The order of the samples was chosen according to the amount of flower-NiMoO_4_ visible in SEM. All XRD analyses were conducted directly on the foam.

X-ray diffraction (XRD) in Figure 2 of the as-synthesized nickel molybdate hydrate on nickel foam confirmed the synthesis of crystalline nickel molybdate hydrate. The diffractograms are in good agreement with previously reported data for nickel molybdate hydrate (JCPDS Card No. 13-0128);^35,54−56^ however the interpretation is challenging. Ghosh et al.^56^ attributed some of the detected peaks in the diffractogram to MoO_3_ but did not detect the MoO_3_ vibrations in their Raman spectrum. Ding et al.^55^ proposed a mixture of NiMoO_4_·1H_2_O and Ni_2_(NO_3_)_2_(OH)_2_·*n*H_2_O or α-Ni(OH)_2_·*n*H_2_O for the diffraction pattern in their work, depending on the pH value during synthesis. Unfortunately, their results are only based on the XRD patterns and no additional characterization method was used to probe the presence of Ni_2_(NO_3_)_2_(OH)_2_·*n*H_2_O or α-Ni(OH)_2_·*n*H_2_O. We do not find any vibrations related to nitrate or nickel hydroxide in our Raman spectra, as shown below. Eda et al.^54^ on the other hand saw similarities in the diffraction pattern of their previous work on CoMoO_4_·^3^/_4_H_2_O with the diffractogram acquired by Ding et al. and proposed a single-phase NiMoO_4_·^3^/_4_H_2_O. So, for basically identical diffraction patterns, the interpretation was quite different. Nevertheless, all these studies related (at least parts of) their diffraction pattern to nickel molybdate hydrate. However, comparing our diffractograms of the as-prepared NiMoO_4_@Nif, we see indication for in fact two different crystal structures among our samples. The peaks at 10°, 14°, 21°, 23°, 24°, 27°, 30°, and 31° show higher intensities for NiMoO_4_@Nif-2 and a decreasing intensity for samples with more flower-NiMoO_4_. We can therefore attribute those peaks to the rod-NiMoO_4_. On the other hand, the peaks at 22°, 34°, 39°, 47°, 53°, and 60° show increasing intensities for samples with more flower-NiMoO_4_ like NiMoO_4_@Nif-5. Hence, we attribute those peaks to the structure predominant in the flower-NiMoO_4_. This means that in contrast to previous publications, we attribute the different diffraction patterns to the different nickel molybdate hydrate nanostructures. The strong peaks detected at 44° and 52° originate from the nickel foam below the catalyst. XRD analysis of the selective Mo etched sample confirms the attribution of the signals to the different structures, as explained later (Figure 5c).

**Figure 3:**
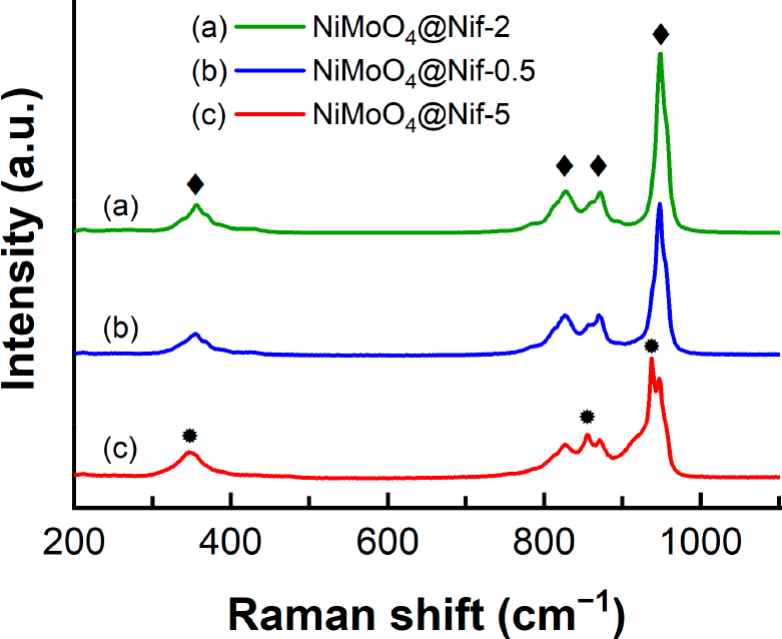
*Ex situ* Raman spectroscopy of the as-synthesized (a) NiMoO_4_@Nif-2, (b) NiMoO_4_@Nif-0.5, and (c) NiMoO_4_@Nif-5 with a 532 nm excitation laser. Vibration modes attributed to rod-NiMoO_4_ are highlighted with “◆”. The vibration modes originating from the flower-NiMoO_4_ are denoted with “●”.

Raman spectroscopy in Figure 3 shows vibration modes that also can be assigned to an overlay of two different nickel molybdate hydrate structures. NiMoO_4_@Nif-2 with only rod-NiMoO_4_ detected in SEM shows a strong peak at 948.0 cm^–1^, which can be assigned to the symmetric Mo=O stretching together with its asymmetric stretching modes at 872.0 and 827.9 cm^–1^ and the Mo–O bending at 356.5 cm^–1^. Additional vibrations modes emerge in NiMoO_4_@Nif-0.5 and are dominant in NiMoO_4_@Nif-5. This second structure shows a strong peak at 938.0 cm^–1^, also assigned to the symmetric Mo=O stretching, a peak at 855.6 cm^–1^, which originates from the asymmetric Mo=O stretching, and a Mo–O bending at 347.9 cm^–1^. All peaks of both structures are in very good agreement with previously reported vibrations for NiMoO_4_·H_2_O.^35,56,57^ On the basis of the amount of observed nanoflowers in the SEM among the different samples, we could attribute the peaks of NiMoO_4_@Nif-2 to the rod-NiMoO4 and the emerging peaks for NiMoO_4_@Nif-0.5 and NiMoO_4_@Nif-5 to the flower-NiMoO_4_. Raman spectroscopy in combination with EDX of selective molybdenum etched samples confirms the assignment of the Raman signals to the different nanostructures (Figure 5b). The different wavenumbers of the symmetric and asymmetric M=O stretching for rod-NiMoO_4_ and flower-NiMoO_4_ indicate a different environment of the molybdenum atom in the two nanostructures, agreeing with different crystallographic phases as proposed by XRD. Extended scans up to 3600 cm^–1^ with very low intensity (down to 0.1%) were also taken with no change of the spectra (SI Figure 10) showing that laser heating effects from the Raman laser can be excluded as a source for structure change.

For attenuated total reflection Fourier transform infrared spectroscopy (ATR-FTIR) investigations, NiMoO_4_ nanostructures were removed from the surface by extensive ultrasonication of NiMoO_4_@Nif in alcohol and analyzed after drying (SI Figure 13). In agreement with Raman spectroscopy, also ATR-FTIR analysis shows signatures that can be rationalized with two structures, with one being more dominant in the NiMoO_4_ from Nif-2 and the second structure showing higher intensities for the NiMoO_4_ from Nif-5. Therefore, we attribute the peaks at 964 cm^–1^, 913 cm^–1^, 884 cm^–1^, 820 cm^–1^, and 733 cm^–1^ to rod-NiMoO_4_, while the ones at 866 cm^–1^ and 690 cm^–1^ and the weak band at 929 cm^–1^ we account to the flower-NiMoO_4_. Those values agree with previous reported vibration wavenumbers for nickel molybdate and polymolybdates.^58−62^ The peak at 913 cm^–1^ is reported for vibrations of molybdates and we assign the peaks at 964 and 884 cm^–1^ to the symmetric and asymmetric Mo=O stretching, the peak at 870 cm^–1^ to the Mo–O–Mo stretching, and the peak at 733 cm^–1^ to the Mo–O bending.

XPS of the as-prepared samples confirmed the EDX result with only nickel, molybdenum, oxygen, and a small amount of carbon being present (SI Figure 14, SI Figure 15, SI Figure 16). The calculated elemental composition based on the area for the elemental analysis (Ni 2p, Mo 3d, O 1s) is displayed in SI Table 2. It also confirms what was previously detected by EDX, namely, an increased Ni:Mo ratio for flower-NiMoO_4_ (Ni:Mo = 1.50 for NiMoO_4_@Nif-5) compared to rod-NiMoO_4_ (Ni:Mo = 1.18 for NiMoO_4_@Nif-2). The nickel 2p_3/2_ and 2p_1/2_ peaks of NiMoO_4_@Nif-2 appeared at 855.9 and 873.9 eV, respectively, which is in very good agreement with previously reported binding energies.^56^ The molybdenum 3d_5/2_ and 3d_3/2_ were observed at 232.3 and 235.5 eV. The oxygen peak could be divided into a lattice oxygen peak at 530.5 eV and a hydroxide part at 532.5 eV, proving the hydrate structure of nickel molybdate.^35^ The lattice oxygen peak could further be divided into Mo–O and Ni–O components,^7^ but a more detailed description of the XPS spectra of the as-prepared sample would have a limited value for this particular study as the material undergoes Mo leaching and phase transitions in the activated catalyst, and it is not considered further. The other samples exhibit similar binding energies (SI Figure 15b, SI Figure 16b).

SEM, EDX, XRD, Raman spectroscopy, ATR-FTIR, and XPS of the as-synthesized materials all agree on a difference in elemental composition and crystallographic phase between the rod-NiMoO_4_ and flower-NiMoO_4_. There are several possible reasons for different structures with various Mo environments, among other poly(oxo)molybdates due to the solution pH,^60^ ratio of molybdenum,^63^ point of zero charge of the substrate,^64^ a solid solution process,^54^ or other molybdates in amorphous or crystalline structures.^54^ Even though no nanoflowers were observed in the SEM analysis of NiMoO_4_@Nif-2, the signals of the flower-NiMoO_4_ detected in Raman spectroscopy, ATR-FTIR, and XRD we believe originate from the dense nanoflower flake structures between the nanorods and the foam, as detected after removing the nanorods by extensive ultrasonication (SI Figure 12a,b).

### Electrochemical Characterization

The as-synthesized material was used as working electrode, and the activity and stability were analyzed with cyclic voltammetry (CV). In the first 20–25 cycles, the peak current density of the Ni^III^/Ni^II^ oxidation at 1.45 V vs RHE (for the first cycle) increased drastically from 15–20 mA cm^–2^ to 120–155 mA cm^–2^, indicating that a large amount of nickel sites became accessible during the activation step, which was further quantified by charge analysis of the oxidation peak (SI Figure 17). After activation the nickel surface coverage was increased by 20–30 times (from 0.38–0.62 μmol cm^–2^ to 11.88–13.99 μmol cm^–2^) (SI Table 3, SI Table 4, SI Table 5). The activation step was detected by time-resolved *in situ* Raman spectroscopy to be the transformation from NiMoO_4_·H_2_O to γ-NiOOH, as described below. Simultaneously with the increase of the Ni^II^ to Ni^III^ oxidation peak current density, the oxidation peak potential shifted to higher values (from 1.45 to 1.53 V vs RHE), while the redox potential at *E*_1/2_ = 1.37 V vs RHE remained constant. This is typical for a non-*iR* compensated curve. The onset of the catalytic wave we detect to be at 1.60–1.65 V vs RHE; hence the increase of current density for higher potentials than the onset potential is due to the oxidation of water to dioxygen. Even though no *iR* compensation was applied, maximum current densities at 1.8 V vs RHE in the range of 210–260 mA cm^–2^ were obtained with overpotentials for 200 mA cm^–2^ between 526 and 563 mV. During the initial stabilization period from the 20th to the 25th cycle to the approximately 200th cycle the maximum current density at 1.8 V vs RHE decreased, reaching 170–190 mA cm^–2^ after stabilization. Those current densities remained during the remaining 300 cycles (≃15 h). Both after activation and after stabilization, NiMoO_4_@Nif-2 demonstrated the best performance among the samples, with decreasing activity for the samples with more flower-NiMoO_4_. This can be rationalized with a slightly lower intrinsic catalytic activity of the surface of nanoflower structure in comparison to the surface of nanorod structure. This is supported by comparing NiMoO_4_@Nif-2 without visible flower-NiMoO_4_ with NiMoO_4_@Nif-0.5 with a small amount of flower-NiMoO_4_ after 500 cycles. Despite having a similar double layer capacitance (*C*_dl_), a lower nickel surface coverage, and a slightly higher solution resistance compared to NiMoO_4_@Nif-0.5, NiMoO_4_@Nif-2 still exhibits a higher current density at 1.8 V vs RHE in the 500th cycle. Tafel plot analyses show slopes of 81–114 mV dec^–1^, which agrees with the hypothesis of different intrinsic activities among the samples and is in agreement with reported values (SI Figure 21).^65^ The loss of maximum current density during the stabilization process we attribute to the loss of surface structures, as visible in the *C*_dl_ plot in the Supporting Information, and not to a deactivation mechanism of the active sites. Worth mentioning is that already during the first anodic sweep, the color of the working electrode changed from bright green to black, which indicates a transformation of the as-synthesized material. The *C*_dl_ of the electrochemical surface area of the samples revealed an increase of the double layer capacitance by covering the bare nickel foam with NiMoO_4_ nanostructures (SI Figure 18). During the activation step, *C*_dl_ further increases 2–3 times after the first 50 cycles, compared to the as-synthesized material. During the subsequent stabilization process it decreased slightly the first 250 cycles, where it finally remained constant at about a factor 2 higher than the initial value for the remaining cycles. We could observe signs of the removal of a few unstable nanostructures from the electrode during the activation step and stabilization process, visible as a very small amount of black particles at the bottom of the electrochemical cell. The analysis of the accumulated double layer capacitance clearly shows that the high activity of the catalyst is mainly due to the marked increase of surface area and the resulting high surface coverage of exposed nickel sites to the electrolyte, formed during the activation step. Furthermore, it shows that the heating ramp during the hydrothermal synthesis has a significant influence on the surface area and therefore also on the nickel surface coverage of the electrode. After the initial activation and stabilization process the high surface area catalysts show a high stability and activity for OER. Electrochemical impedance spectroscopy (EIS) revealed a slight increase of solution resistance (high-frequency resistance) during the 500 cycles from 2.47–2.62 Ω to 2.72–3.1 Ω (SI Figure 20), which could be due to the formation of a more porous structure.

Care also needs be taken with a correct estimation of the surface area, as recently pointed out by Zheng et al.,^66^ addressing the overestimation of catalytic activity on foam type electrodes due to normalizing of the current to a wrong surface area. However, since we are not focusing on the intrinsic activity of our catalyst, we normalize the current density to the 2D projected area of the electrode, if not indicated differently, as the effect of increase in surface area has been addressed in our analysis of double layer capacitance above. For a microscopic normalization, we instead refer to the Supporting Information where a graph is normalized to the geometric surface area of the bare foam (SI Figure 22).

### Postelectrochemical Characterization of the Samples

SEM analysis of the catalyst after catalysis corroborates the apparent high stability of the nanostructures after its activation step and stabilization process (SI Figure 23, SI Figure 25, SI Figure 26). Even after more than 25 h of cyclic voltammetry the nanorod and nanoflower structures were still present. Some nanorods seemed to be broken and fused together. Interestingly all nanorods obtained a roughened surface, indicating a contribution to the increased *C*_dl_. The diameters of those nanorods remained in the same range as before electrochemistry. The nanoflower structure seemed unchanged. The macrostructure, however, revealed a more open porous structure with more spherical larger and smaller pores, possible due to the removal of nanostructures by oxygen bubbles or molybdenum leaching, as explained later. EDX analysis of the sample revealed loss of molybdenum in the material and the presence of potassium homogeneously dispersed all over the structures (SI Figure 24).

Surface analysis using XPS on NiMoO_4_@Nif-2 after electrochemistry (SI Figure 27) revealed what was already detected by the deeper probing EDX, i.e., that only a small amount of molybdenum remained in the material. Instead we confirm the presence of potassium, which we will explain below. Nickel 2p_3/2_ and 2p_1/2_ remained basically unchanged at 855.5 and 873.2 eV, respectively, consistent with nickel hydroxide and nickel oxyhydroxide.^67,68^ The oxygen 1s peak shows contributions from three different oxygen environments. One small and broad peak is detected at 532.3 eV from oxygen in water. A large peak at 530.8 eV is attributed to hydroxide, whereas the peak at 529.0 eV is from metal oxide. The oxygen 1s spectrum showed a highly increased amount of hydroxide (82.2%) compared to metal oxide (15.7%). Excluding the contribution of oxygen in water (2.1%), we calculated the atomic ratio to be 33.9 atom % nickel, 65.9 atom % oxygen, and only 0.2 atom % molybdenum. It is in good agreement with the other samples (SI Figure 28, SI Figure 29, SI Table 6).

To quantify the loss of Mo in the catalyst, inductively coupled plasma optical emission spectrometry (ICP-OES) was utilized to measure the presence of molybdenum in the electrolyte after 500 cycles. We found a mass concentration of 36.984 mg L^–1^ (RSD 0.8%), 24.868 mg L^–1^ (RSD 0.6%), and 28.389 mg L^–1^ (RSD 0.4%) for NiMoO_4_@Nif-2, NiMoO_4_@Nif-0.5, and NiMoO_4_@Ni-5, respectively. From the acquired ICP-OES data we confirm that all materials have a significant molybdenum leaching into the electrolyte.

At this point we can summarize that during electrochemistry, our as-synthesized nickel molybdate hydrate experiences substantial molybdenum leaching into the electrolyte, which increases the amount of accessible nickel sites.

### Time Resolved *in Situ* and *Operando* Raman Spectroscopy

**Figure 4:**
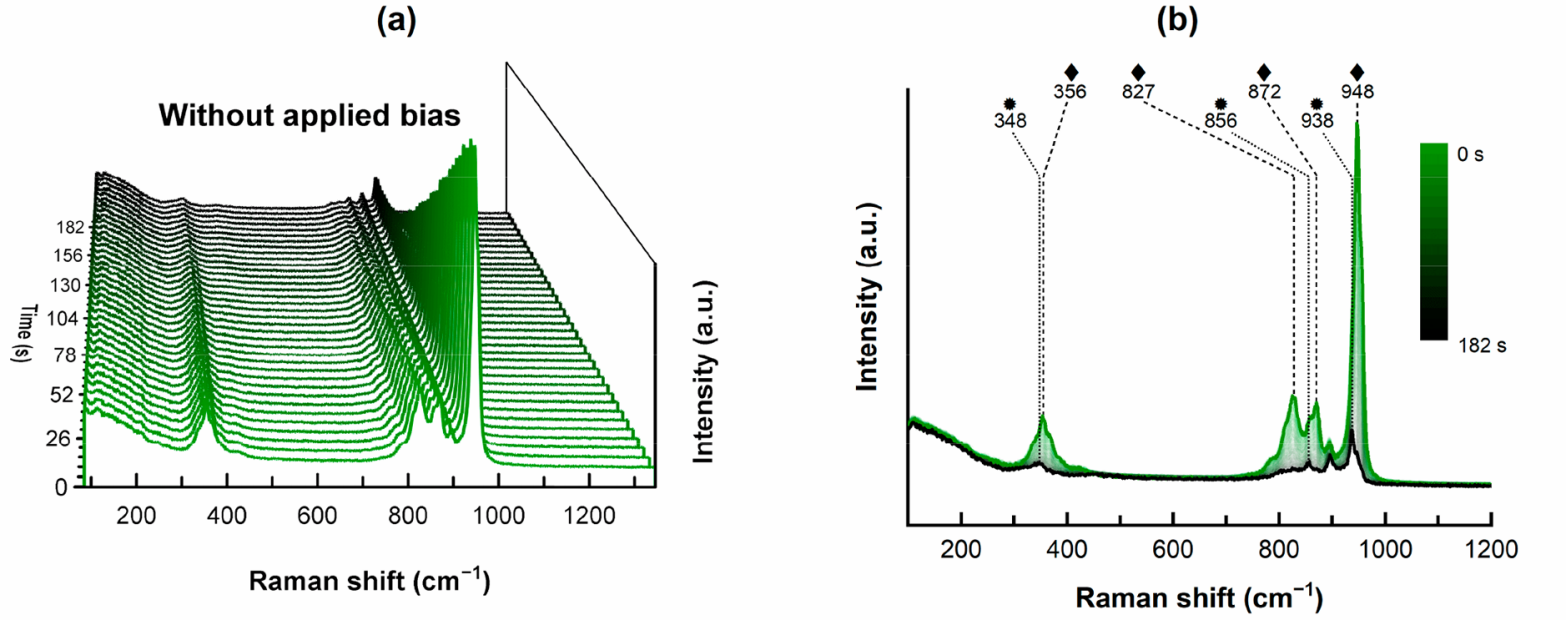
Time resolved *in situ* Raman spectroscopy without applied bias of NiMoO_4_@Nif-2 in 1.0 M KOH over 180 s. We acquired the spectra with 50% laser intensity for 5 s subsequently without any quiet time in between. (a) 3D plot of the single spectra with the first acquisition at the very front and the last acquisition after 182 s at the very end. (b) The same data illustrated in a 2D plot, showing the removal of the rod-NiMoO_4_ vibration features (◆), and only the vibrations of flower-NiMoO_4_ (●) remains. The vibration at 896 cm^–1^ from the monomolybdate in solution is not further discussed.

**Figure 5:**
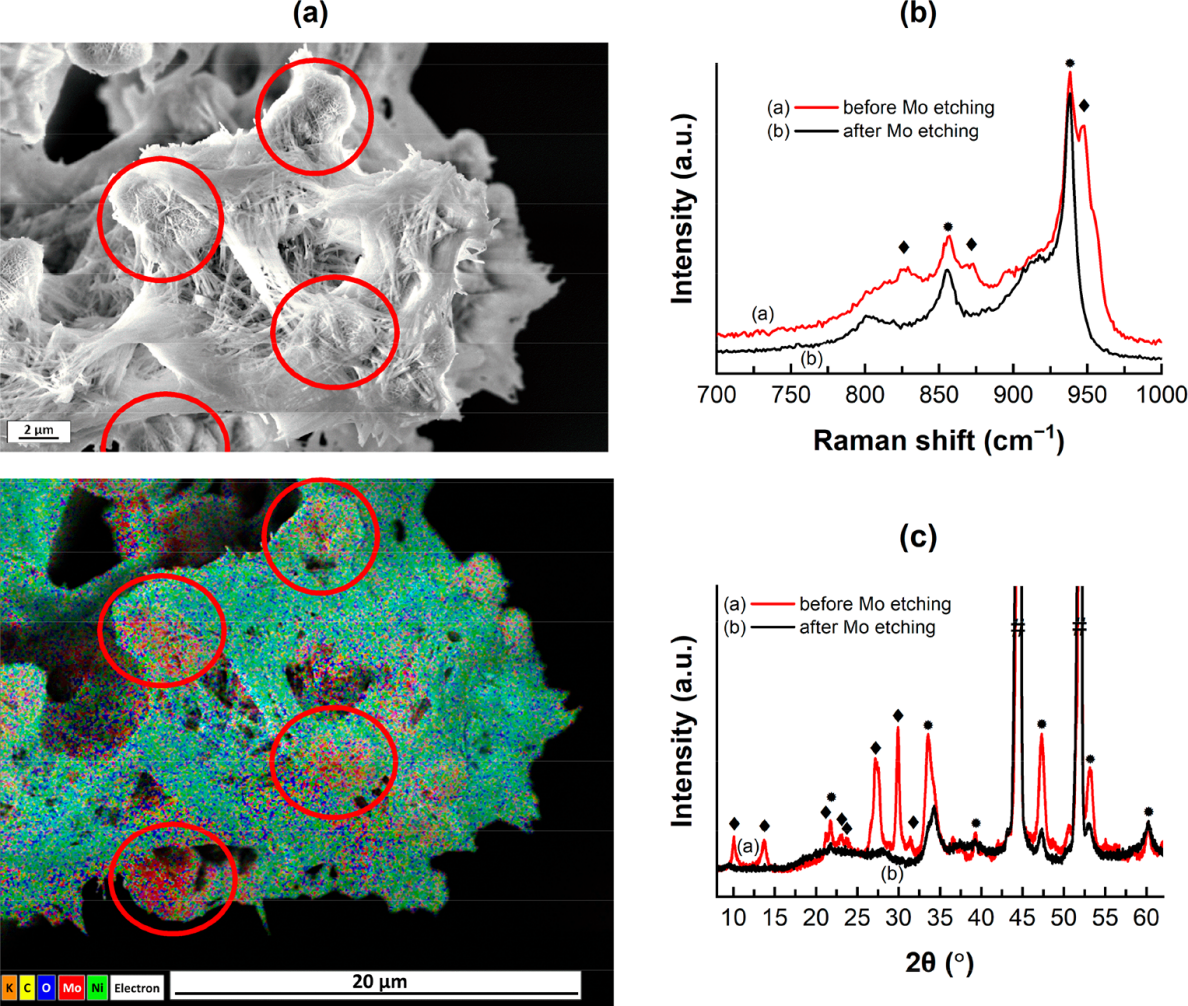
Selective molybdenum etching enables verification of Raman vibration and XRD pattern assignment to different NiMoO_4_ nanostructures. (a) Secondary electron (SE) image (top) of NiMoO_4_@Nif-5 after selective molybdenum etching with clear presence of nanorod and nanoflower structures. On the bottom is the EDX mapping of the same area, showing that molybdenum (red) is only present in the nanoflower structure after molybdenum etching. Red circles indicate some areas with nanoflowers in the SE image and the corresponding positions in the EDX mapping. Oxygen (blue) and nickel (green) are still present in both nanostructures. (b) Raman spectra focused on the stretching modes of Mo=O in NiMoO_4_@Nif-5 before (red) and after (black) molybdenum etching from the nanorod. The red spectrum has both vibrations from rod-NiMoO_4_ (◆) and flower-NiMoO_4_ (●). The black spectrum shows only vibrations from flower-NiMoO_4_. (c) XRD of the same sample before (red) and after (black) selective molybdenum etching, illustrating the removal of the rod-NiMoO_4_ (◆) diffractions during the process.

**Figure 6:**
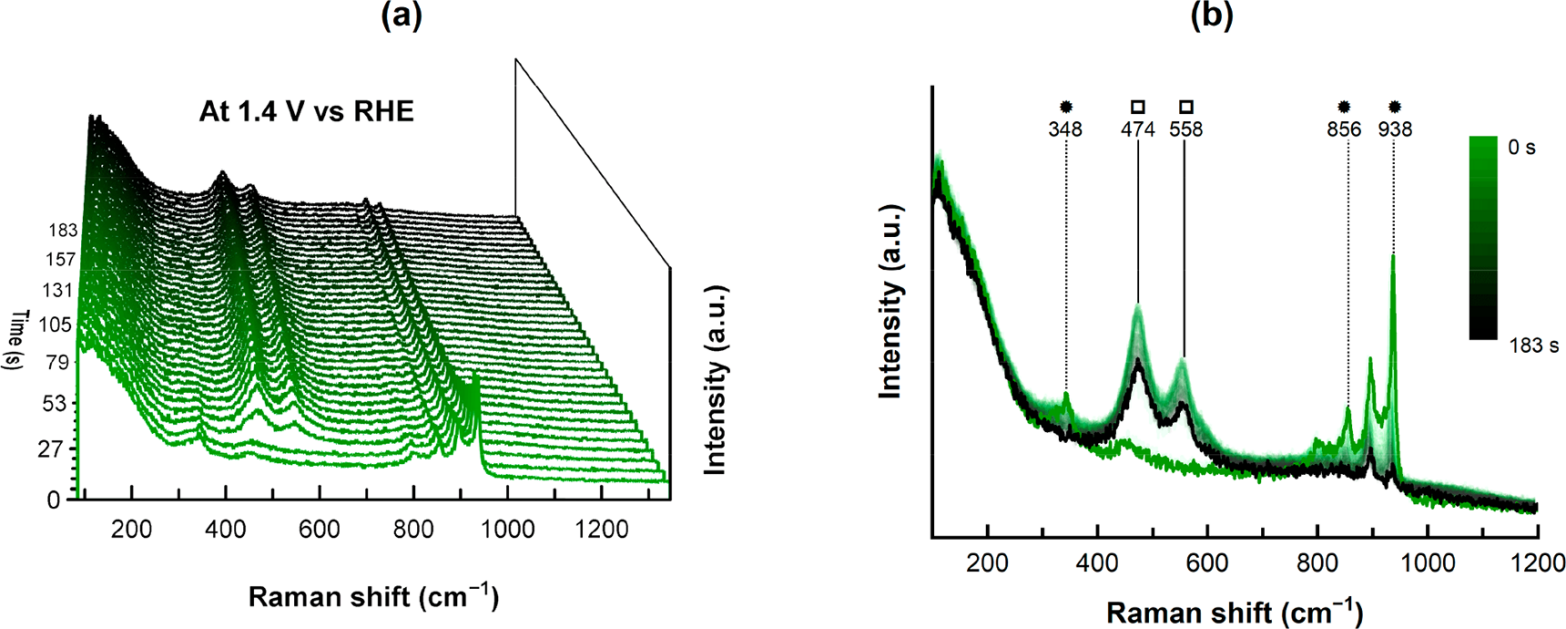
Time resolved *in situ* Raman spectroscopy at 1.4 V vs RHE of NiMoO_4_@Nif-2. (a) 3D plot of the single spectra with the first acquisition at the very front and the last acquisition after 183 s at the very end. (b) The same data illustrated in a 2D plot illustrating the sudden removal of the flower-NiMoO_4_ (●) vibration signals and the instantaneous formation of γ-NiOOH (□). The signals from Ni(OH)_2_ at 450 cm^–1^ and molybdate in solution at 896 cm^–1^ are not highlighted.

**Figure 7:**
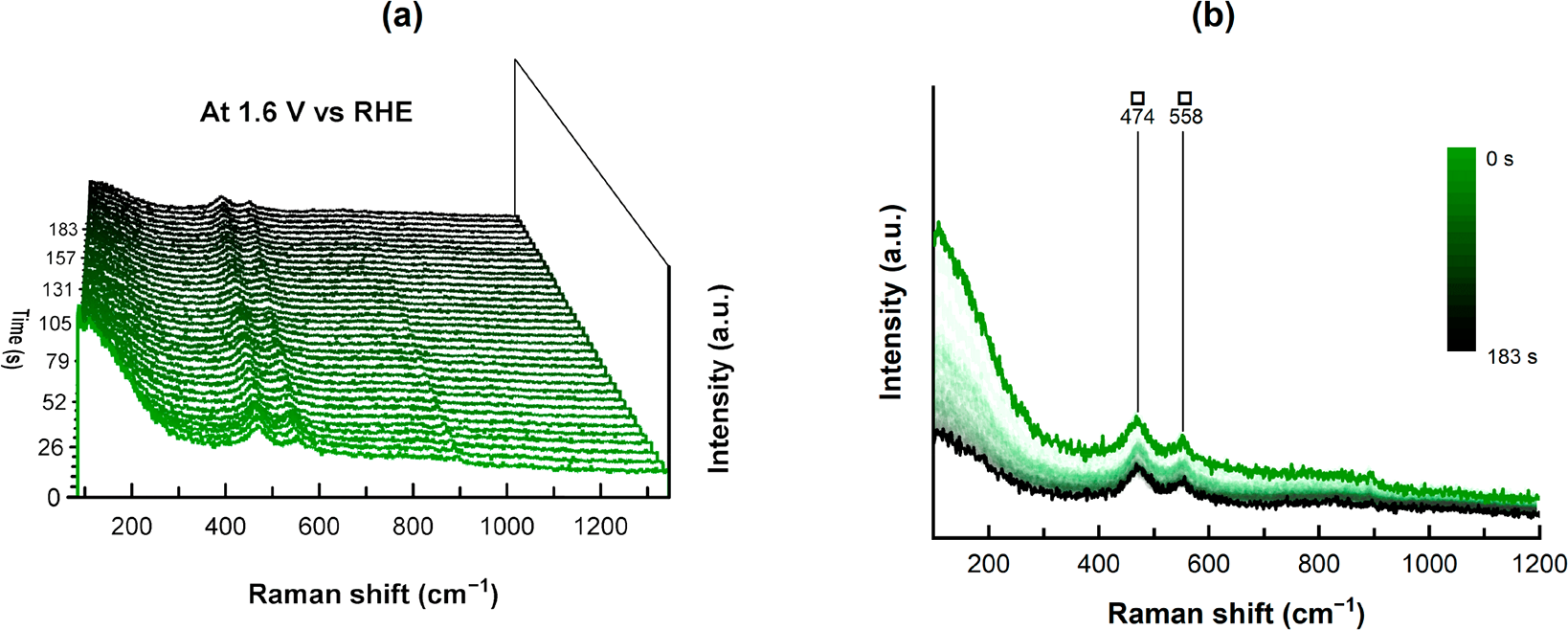
Time resolved *operando* Raman spectroscopy at 1.6 V vs RHE of NiMoO_4_@Nif-2. (a) 3D plot of the single spectra with the first acquisition at the very front and the last acquisition after 183 s at the very back. (b) The same data illustrated in a 2D plot, displaying the presence of majorly γ-NiOOH vibrations (□). The small signal at 896 cm^–1^ is from a monomolybdate in solution.

We here use the notion of *in situ* for measurements of the catalyst material immersed under the electrolyte and at applied potentials not yet starting the OER, while *operando* refers to measurements under the OER process and O_2_ generation. Time resolved *in situ* Raman spectroscopy in 1.0 M KOH without any applied bias unveiled the instantaneous disappearance of the rod-NiMoO_4_ vibrations at 948 cm^–1^, 872 cm^–1^, 827 cm^–1^, and 356 cm^–1^, caused by molybdenum etching, whereas the flower-NiMoO_4_ vibration signals at 938 cm^–1^, 856 cm^–1^, and 348 cm^–1^ remained stable (Figure 4, SI Figure 32a, SI Figure 33a). This enabled us to also assess the stability of the different nickel molybdate hydrate structures in 1.0 M KOH. As presented in the section before, EDX and XPS indicate with the loss of molybdenum and oxygen during the 500 cycles a removal of a molybdenum oxide (SI Figure 24f, SI Table 6). However, since we did not unambiguously detect the leaching-out species for low number of cycles, we continue to call this process “molybdenum etching”. This result also explains the additional peak detected at 896 cm^–1^, which is attributed to a monomolybdate in the electrolyte. Nevertheless, this selective molybdenum etching provided us with the opportunity to verify the assignment of the different vibration signals and diffraction patterns to the different nanostructures by EDX mapping and XRD analysis of an etched sample (Figure 5), which was until then only an assumption. Important to realize is that Raman spectroscopy only revealed the instability of the nickel molybdate hydrate compound. The nanorod morphology is still present, even though the rod-NiMoO_4_ vibrations vanished. The same is also the case for the flower-like shaped nanostructure and the flower-NiMoO_4_ vibrations later on. Consistent with the *in situ* measurements without applied bias, the flower-NiMoO_4_ vibrations remained unchanged also during the time-resolved *in situ* Raman measurement in the nonfaradaic regime by applying a bias of 0.95 V vs RHE via controlled potential electrolysis (CPE) (SI Figure 31b, SI Figure 32b, SI Figure 33b). This corroborates its higher stability in 1.0 M KOH compared to rod-NiMoO_4_. Only a small peak at approximately 450 cm^–1^ appeared, which could be from Ni(OH)_2_,^69^ possibly formed in a subsequent surface reaction of the remaining nickel or nickel oxide from the nanorods with the electrolyte after the molybdenum etching. At 1.4 V vs RHE the vibration of the flower-NiMoO_4_ signal vanishes with the Ni^II^ to Ni^III^ oxidation, simultaneous with the immediate emergence of γ-NiOOH signals at approximately 474 and 558 cm^–1^ (Figure 6, SI Figure 32c, SI Figure 33c), which correspond to the bending and stretching vibration modes of Ni–O, respectively. The detected wavenumbers are in good agreement with reported values for this phase.^27,34,70^ From the unchanged signal during time-resolved *operando* Raman spectroscopy at 1.6 V vs RHE in Figure 7 we conclude that there is no further transformation of the catalyst during catalysis and that γ-NiOOH is the actual catalyst for OER. This is further corroborated with time-resolved *operando* Raman spectra at even higher potentials (up to 1.8 V vs RHE) (SI Figure 31f,g, SI Figure 32e–g, SI Figure 33e–g). The variation of intensities in the time-resolved *operando* Raman spectra for potentials at 1.6 V vs RHE and above are due to evolved oxygen gas bubbles scattering the light (SI Figure 31e–g, SI Figure 32e–g, SI Figure 33e–g).

γ-NiOOH is a well known and highly efficient oxygen evolution catalyst,^27,71,72^ which is often further improved by iron doping.^34,73,74^ Since the CPE at 1.4 V vs RHE forms the active site for water splitting, we cannot exclude any oxygen evolution reaction happening already at this potential. Extrapolating the voltammograms in SI Figure 17 indicates already minor activity toward OER at this potential. However, since the Ni^II^ to Ni^III^ oxidation wave overlaps with a possible catalytic wave in this potential range and the actual obtained current from water splitting would be marginal, we refrain from calling Raman spectroscopy at this potential “*operando*” and stay with the more conservative “*in situ*”. We conclude that the activation step detected in CV is the formation of γ-NiOOH from the NiMoO_4_·H_2_O precatalyst, which occurs via two different processes. For the rod-NiMoO_4_, molybdenum immediately leaches out when in contact with the electrolyte, leaving a nanorod scaffold of oxygen and nickel behind. In a subsequent reaction upon Ni^II^ to Ni^III^ oxidation this scaffold reacts with the electrolyte to form γ-NiOOH. For the flower-NiMoO_4_ it is unclear if the molybdenum leaching and γ-NiOOH formation happen simultaneously or subsequently as for the rod-NiMoO_4_. Since we do not see any new signal emerging in the Raman spectrum during the transformation, we exclude an intermediate step via Ni(OH)_2_. Since γ-NiOOH catalyzes the OER, a fast γ-NiOOH formation is desired. Not only does rod-NiMoO_4_ provide a larger surface area than flower-NiMoO_4_, but due to the rapid molybdenum leaching in the electrolyte, it readily also transforms to γ-NiOOH upon the Ni^II^ to Ni^III^ oxidation. During the revision of this paper we found that very recently, Choi et al. reported an interesting and comprehensive study with similar findings on the formation of γ-NiOOH, starting with nickel molybdate nanorods.^65^ They also detected different molybdate Raman vibrations for *ex situ* and *in situ* in 1 M KOH but attributed the new spectra to the dissolution of molybdenum into the electrolyte and not to remaining molybdate in the nanoflowers form as revealed in our study. Their observed molybdate signal for low applied potentials and its removal due to the nickel oxidation agree with the results in our work. There is also a related work focusing on molybdenum leaching in Mo containing metal oxides during catalysis, increasing the number of active sites.^38^

The irreversible transformation from NiMoO_4_·H_2_O to γ-NiOOH becomes obvious with the time-resolved *in situ* Raman spectra after all CPE, in which the signal from γ-NiOOH remains stable and no indication for an emerging NiMoO_4_·H_2_O vibration is detected. All *in situ* and *operando* spectra and their corresponding controlled potential electrolysis (CPE) responses are shown in SI Figures 31–34. After the selective molybdenum etching no rough structure on the nanorods was detected. Therefore, we assume that the roughening of the nanorod structures is caused by the formation of γ-NiOOH. Furthermore, potassium from the electrolyte (KOH) is known to intercalate in γ-NiOOH,^72^ which explains the detection of potassium in XPS and EDX after 500 cycles in SI Figure 24 and SI Figures 27a–29a. The characteristic peak for γ-NiOOH in the Ni Auger spectrum is not detected in XPS after electrochemistry. Since the XPS measurement was not conducted directly after the electrochemical exposure, we believe that with time γ-NiOOH reduced to Ni(OH)_2_, which was then detected in XPS. This would agree with the Ni 2p binding energies,^67^ the spin-energy separation of 17.7 eV, which is close to previously reported values,^35,75,76^ and the detected Ni:O ratio of 1:1.95.

### Monitoring Molybdenum Etching with ICP-OES

Exchanging the electrolyte between each *in situ* and *operando* Raman measurement would risk a change of the spot, on which Raman spectroscopy was conducted, due to possibilities of movement on the micrometer scale. Hence, CPE was repeated with a second sample at the same potentials and duration as for the time-resolved *in situ* and *operando* Raman spectroscopy measurements. With that we can correlate the molybdenum leaching detected by ICP-OES in the electrolyte with the acquired time-resolved *in situ* and *operando* Raman spectra (SI Figure 35, SI Table 7). From the electrolytes, in which the flower-NiMoO_4_ remained stable (without applied bias and at 0.95 V vs RHE) the molybdenum concentrations follow the trend of the amount of rod-NiMoO_4_, from which this molybdenum was leached. NiMoO_4_@Nif-2 shows the highest concentration, while NiMoO_4_@Nif-5 shows the lowest at a similar working electrode size. The high concentration of molybdenum during the CPE at 0.95 V vs RHE we attribute to the leaching of remaining rod-NiMoO_4_. The low molybdenum concentration after CPE at 1.4 V vs RHE and higher potentials lead to two important interconnected conclusions. First, molybdenum of the rod-NiMoO_4_ seems to be completely leached out, as otherwise the concentration of molybdenum needed to be higher, as seen for 0.95 V vs RHE. Second, the molybdenum leaching rate from the nanoflower structure is very low. Recalling that XPS and EDX showed that the molybdenum concentration of the as-prepared flower-NiMoO_4_ was lower compared to the rod-NiMoO_4_, a slightly lower Mo leaching is expected when leaching comes from the flower-NiMoO_4_. The prolonged presence of the vibrational features for the flower-NiMoO_4_ during *operando* Raman corroborates the slower leaching process. With EDX we still detected some molybdenum in the nanoflower shapes after CPE (SI Figure 36). This in combination with the XPS result after 500 cycles could explain why there are different reports on the stability of NiMoO_4_ and the presence of molybdenum after electrochemistry. As comparison, the samples that were used for CPE were tested for in total 18 min, whereas the samples that were used for the CV test were tested for more than 25 h.

Apart from the applied potential in the electrolysis and the surrounding alkaline environment driving NiMoO_4_ into γ-NiOOH, the formation of the two different structures of NiMoO_4_ is also interesting as this enables control and selectivity of a desired structure. Here, the heating rate during the hydrothermal synthesis has a strong influence as we have shown in this work. Using the time-dependent *in situ* Raman spectroscopy results and structure stability of the different nanostructures in 1 M KOH, we propose that the rod-NiMoO_4_ structures are being built up from larger polymolybdate oxoanions, while the aggregated flake-like structures in the flower-NiMoO_4_ are being built up from smaller molybdate oxoanions. Our molybdate source is a monomolybdate; however, in the pH regime of our hydrothermal synthesis solution a heptamolybdate is reported to be more stable.^64^ This could also explain why the first layers on the nickel foam are composed of nanoflowers and dense flakes. First, the polymolybdate formation may take time, resulting in that only monomolybdates or smaller molybdate anions are available for forming the nickel molybdate hydrate on the first layers of the foam at the pH used in the synthesis. Second, a study from Kim et al. found that the molybdate species on the first layer on the foam is depending on the point of zero charge (PZC) of the substrate. Nickel oxide has a PZC of 10.5, which is in the pH regime in which the monomolybdate species is stable.^33,64^ Furthermore, larger and smaller molybdate oxoanions could explain the molybdenum leaching of rod-NiMoO_4_ in 1 M KOH, in which larger polymolybdates like heptamolybdate anions are not stable. On the other hand, the flower-NiMoO_4_ remains stable, since the monomolybdate is stable under that condition. A further indicator for smaller and larger polymolybdates can be found in the Raman spectra. The value for the symmetric Mo=O stretching at 948.0 cm^–1^ was also reported in the works of Kim et al.^64^ and Dobrea et al.^63^ for the same vibration mode in heptamolybdate. Additionally they described a shift of the same vibrations to lower wavenumbers for smaller molybdates, which fits with the symmetric Mo=O stretching for the flower-NiMoO_4_ at 938.0 cm^–1^.^63,64^ Moreover, a larger polymolybdate participating in the formation of nanorod NiMoO_4_ structure could explain why the molybdenum content in the nanorods is higher compared to the nanoflowers, as detected by elemental analysis.

## Conclusions

The development and evaluation of nanodimensional catalysts are important for progress of the field and for the final application. For further improvement of the catalytic activity and a deeper understanding of the material and its active phase it is essential to elucidate the contribution of the different added elements. Therefore, it is vital to investigate the catalyst in operation, since the reaction conditions are substantially different from the conditions under which they were synthesized, and dynamical changes and phase transformations of the material can occur. In this work we report that NiMoO_4_·H_2_O is transformed into γ-NiOOH as the active catalyst for water oxidation in alkaline media, where molybdenum only works as a morphology inducing agent and is not retained in the active catalyst. First, molybdenum acts as a structure former, building up different nanostructures to increase the surface area. In a second step molybdenum leaches out of the as-synthesized material, and with the oxidation of Ni^II^ to Ni^III^ in 1.0 M KOH, a high surface γ-NiOOH catalyst with a drastically increased number of accessible nickel sites is formed. The leaching occurs slightly differently and with different rates depending on the exact composition and structure of the NiMoO_4_ and could explain the previously different conclusions reported from this system. The molybdenum of rod-NiMoO_4_ already leaches out in 1.0 M KOH before the Ni^III^/Ni^II^ oxidation. NiMoO_4_ nanostructures in aggregated flakes (flower morphology) are in comparison more stable in the electrolyte and lose their molybdenum upon oxidation of Ni^II^ to Ni^III^. Molybdenum leaching and the simultaneously formation of γ-NiOOH, which is also correlated with the roughened surface of the nanorods, lead to a high surface area. The different shapes of the nickel molybdate hydrate nanostructures can be controlled by different heating ramps during the hydrothermal synthesis and can be already distinguished by their Raman and ATR-FTIR spectra and XRD diffraction pattern. The results can thus explain why previous studies have come to different conclusions, where the apparent stability would depend on the synthesis route as well as the time period and current density used in the stability tests. We show that the different crystal structure and nanostructure likely originate from larger and smaller polymolybdate oxoanions building up the nickel molybdate hydrate. This is supported by different stabilities and by the shift of the symmetric Mo=O vibration mode observed in our Raman measurements, a vibration known to be shifted to lower wavenumbers for smaller molybdates. The results show that a detailed investigation of structural changes by comparing *in situ* and *operando* spectroscopies can shed light on the dynamic formation of active catalyst phases and their stability. Furthermore, the local structural difference in rod and flower shaped nanostructures in the investigated system can be analyzed and can give insight into the metal molybdate hydrate nanostructure formation and stability. It is anticipated that a similar approach can be applied to many other nanostructured catalyst systems, unveiling their formation, structural integrity, and dynamic reformulation into the active catalyst phase.

## Methods

### Materials

All precursor materials are used as received without further purification. Nickel(II) nitrate hexahydrate (purum p.a., crystallized, ≥97.0% (KT)) and sodium molybdate dihydrate (ACS reagent, ≥99%), ultrapure water (for UHPLC, for mass spectroscopy), potassium hydroxide (pellets for analysis, EMSURE), and fuming hydrochloric acid (37%, for analysis, EMSURE) were provided by Sigma-Aldrich. Ethanol absolute (AnalaR NORMAPUR ACS, Reag. Ph.Eur. for analysis, ≥99.8%) and 2-propanol (GPR RECTAPUR, ≥99.0%) was used from VWR Chemicals. Nickel foam (800 μm pore size, 2.6 mm thickness, 90% porosity, 6.0 m^2^ L^–1^ geometric surface area, 99.9% purity) was provided by Alantum Europe GmbH. Deionized water was supplied by the universities purification system. For ICP-OES the molybdenum single element standard solution (1000 μg L^–1^ in H_2_O) and water (ASTM type I, 18 MΩ) was purchased from Lab Analytical, PerkinElmer.

### Synthesis

Nickel molybdate hydrate was synthesized on nickel foam with a hydrothermal synthesis, inspired by Wang et al. and Zhang et al.^7,35^ In short, a cleaned nickel foam was added to a 60 mL aqueous solution of 0.04 M nickel nitrate hexahydrate and 0.04 M sodium molybdate dihydrate in a Teflon lined stainless steel autoclave and heated up to 150 °C with heating ramps of 0.5 °C min^–1^, 2 °C min^–1^, and 5 °C min^–1^. All samples were held at 150 °C for 6 h and cooled down naturally. The as-synthesized nickel molybdate hydrate on nickel foam was denoted according to their heating ramps as NiMoO_4_@Nif-0.5, NiMoO_4_@Nif-2, and NiMoO_4_@Nif-5, respectively. A more detailed description of the synthesis is given in the Supporting Information.

### Characterization

#### Scanning Electron Microscopy and Energy Dispersive X-ray Spectroscopy

For the analysis of the nanostructure a high-resolution scanning electron microscope (SEM) (ZEISS 1530) with a Schottky FEG and acceleration voltages 2–10 kV was used. An Inlens secondary electron detector was used for imaging. Energy dispersive X-rays (EDX) were detected with Oxford Instruments X-Max^N^ and analyzed with the corresponding AZTec software.

#### X-ray Diffraction

X-ray diffraction (XRD) of the as-synthesized NiMoO_4_@Nif was done using a Bruker D8 Advance diffractometer (solid state rapid LynxEye detector, Cu Kα radiation, Bragg–Bretano geometry, DIFFRACT plus software) between 5° and 90° 2θ with at step size of 0.013°. The time per step was set to 4 s. All diffractograms were acquired at room temperature. The samples were immobilized on a sample holder and their height was adjusted, in order to compare the intensities of the different patterns. The collected data were analyzed with the HighScore Plus 3.0 software from PANalytical.

#### Raman Spectroscopy

Raman spectroscopy was conducted with a Renishaw Reflex (Invia) Raman spectrometer with a frequency doubled Nd: YAG 532 nm laser beam, a grating of 2400 lines mm^–1^, and a Renishaw streamline CCD 1024 chip detector. Prior to experiments, the Raman spectrometer was calibrated with a silicon reference to (520.5 ± 0.2) cm^–1^. For all measurements the software WiRE 3.4 was used. A more detailed description of the setup for the different modes (*ex situ*, *in situ*, *operando*) is given in the Supporting Information.

#### X-ray Photoelectron Spectroscopy

X-ray photoelectron spectroscopy (XPS) measurements was performed with a Physical Electronics PHI Quantera II scanning XPS microprobe with monochromatic Al Kα X-rays with 1486.6 eV and acceleration voltage of 15 kV. For the survey spectra an X-ray beam diameter of 200 μm with a power of 50 W was used with a pass energy of 224.00 eV and 100 ms per step. For the high-resolution elemental analysis, the X-ray beam diameter was decreased to 100 μm and the pass energy to 55.00 eV. All spectra were analyzed with CasaXPS software^77,78^ while using the R.S.F. values from the PHI MultiPak,^79^ the software for PHI Quantera II. For elemental analysis a Shirley background correction was applied. All spectra were charge corrected versus adventitious carbon at 284.8 eV binding energy.

#### Electrochemical Characterization

All electrochemical measurements (CV, EIS, CPE) were done with a CHI 760C potentiostat from CH Instruments and the corresponding software in a three-compartment cell with a platinum wire or mesh as counter electrode (CE) and a Ag/AgCl (sat. KCl) reference electrode (RE). The working electrode (WE) was the synthesized NiMoO_4_@Nif clamped in a PTFE sample holder, which was fully immersed into the 1.0 M KOH electrolyte. All potentials are reported versus reversible hydrogen electrode (RHE) and calculated according to eq 1.
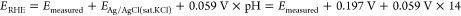
1

For the cyclic voltammetry to investigate the catalytic activity, the WE was cycled between 0.9 and 1.8 V vs RHE with 10 mV s^–1^ and no *iR* compensation. The surface coverage (Γ) of nickel sites was calculated by analyzing the Ni^III^/Ni^II^ oxidation peak following eq SI 2. The double layer capacitance (*C*_dl_) was calculated based on the nonfaradaic current density at 0.95 V vs RHE for different scan rates. Electrochemical impedance spectroscopy was obtained by the alternating current impedance technique sweeping between 0.001 and 100 000 Hz with an amplitude of 5 mV at 1.25 V vs RHE. For the *in situ* and *operando* Raman spectroscopy analysis a fixed potential (0.95, 1.4, 1.5, 1.6, 1.7, 1.8 V vs RHE) was applied for 180 s via controlled potential electrolysis. More detailed information is given in the Supporting Information.

#### Inductively Coupled Plasma Optical Emission Spectrometry

For ICP-OES analysis an Avio 200 model from PerkinElmer was used with the Syngistix software. During ICP-OES the argon gas flow was set to 8 L min^–1^ for the plasma, 0.2 L min^–1^ for the auxiliary, and 0.7 L min^–1^ for the nebulizer. The radiofrequency power (RF) of the plasma was 1500 W. The pump flow rate was 1.00 mL min^–1^. Descriptions of the different solutions and their preparation are displayed in the Supporting Information.

## Supporting Information Available

More detailed explanation of the synthesis procedure and characterization methods; SEM/EDX of the as-synthesized NiMoO_4_@Nif; *ex situ* Raman spectra with different intensities and extended scans; *ex situ* Raman spectra of the removed NiMoO_4_ particles for ATR-FTIR; SEM and *ex situ* Raman investigation of the extensive ultrasonicated NiMoO_4_@Nif-2; ATR-FTIR of the removed NiMoO_4_ nanoparticles; XPS analysis of the as-synthesized NiMoO_4_@Nif; EIS before and after 500 cycles; CV with 500 cycles; calculation of nickel surface coverage; determination of *C*_dl_ and its dependence on the range of scan rates; CV normalized to the geometric surface area of the bare nickel foam; SEM, EDX, and XPS analysis of the material after 500 cycles; secondary electron imaging and EDX mapping after selective molybdenum etching; time resolved *in situ* and *operando* Raman spectra and the corresponding CPE; ICP-OES after CPE; EDX analysis of NiMoO_4_@Nif-5 after CPE (PDF)
