# Correction to “From NiMoO_4_ to γ-NiOOH: Detecting the Active Catalyst Phase by Time Resolved *in Situ* and *Operando* Raman Spectroscopy”

**DOI:** 10.1021/acsnano.1c10145

**Published:** 2021-11-30

**Authors:** Robin N. Dürr, Pierfrancesco Maltoni, Haining Tian, Bruno Jousselme, Leif Hammarström, Tomas Edvinsson

After our article was published we became aware of the comprehensive and enlightening study by Liu et al.,^1^ which we would like to accentuate. In their work, they detect the complete reconstruction of NiMoO_4_·*x*H_2_O nanorods into a highly porous and loose γ-NiOOH structure by electrooxidation in 1 M KOH. By high-resolution transmission electron microscopy (HRTEM) and electron tomography analysis, they could observe that molybdenum leaching, before the oxidation of Ni^2+^ to Ni^3+^, causes an amorphous Ni–O layer. This agrees with our X-ray diffraction (XRD) data after molybdenum etching, in which no crystalline phase other than the one of flower-NiMoO_4_ could be detected.^2^ By HRTEM, they could infer that the formed γ-NiOOH nanorods are built up from nanosheets when the etching and oxidation step occur subsequently and not simultaneously, which confirms our observation of a roughened sheet-like morphology of our nanorods after catalysis. As in our work, the removal of the vibration spectra of the nanorods was detected by time-resolved *in situ* Raman spectroscopy measured without applied bias. However, in contrast with us, they suggest that it is the vibration environment that is responsible for the shift of the peak at 355 cm^–1^ to lower wavenumbers, whereas in our work, this lower wavenumber is assigned to the presence of a flower-NiMoO_4_ sheet structure between the NiMoO_4_·*x*H_2_O nanorod structure and nickel foam. This was confirmed in our study by performing complementary XRD and Raman spectroscopy studies of flower-NiMoO_4_ and NiMoO_4_ nanorods by both selective etching and the additional synthesis of samples with domination of one of the allotropes. We also observe a shift of the peak at 948 cm^–1^ to slightly lower wavenumbers in their spectra, which, again, is consistent with presence of flower-NiMoO_4_ sheet structures between the rods and the foam. Interestingly, the anhydrous form of NiMoO_4_·*x*H_2_O, which is also known as α-NiMoO_4_, shows a much slower leaching rate in 1 M KOH compared with the nanorod-shaped NiMoO_4_·*x*H_2_O.^3^ Eventually, with 30 wt % KOH or an increased temperature to 51.9 °C in 1 M KOH (as shown in an adjacent study^3^), molybdenum leaching was achieved for α-NiMoO_4_. They attributed this to a very limited molybdenum leaching rate that was accelerated by higher concentrated KOH or temperature.^2^ With the same reasoning and instead considering two different crystal structures, one with more dense/closer packed Ni atoms, it would agree with our detected different molybdenum leaching rates among the different nanostructures, which also possess different crystal structures. This addendum is meant to highlight and acknowledge some recent work we missed in our contribution, with the intention that the additional comments and comparisons made here bring a more complete understanding of the structures and processes present in these systems.
