# Supplementary material for: From NiMoO4 to γ-NiOOH: Detecting the Active Catalyst Phase by Time Resolved in Situ and Operando Raman Spectroscopy
Source: ACS Nano. 2021 Aug 12;15(8):13504–15. doi: 10.1021/acsnano.1c04126 (PMC8388116; doi:10.1021/acsnano.1c04126)
Supplement: Supplementary file 1 — nn1c04126_si_001.pdf [file nn1c04126_si_001.pdf]

Supporting Information for

From $NiMoO_4$ to γ-NiOOH - Detecting the Active Catalyst Phase by Time Resolved *In Situ* and *Operando* Raman Spectroscopy

Robin N. Dürr [a], Pierfrancesco Maltoni [b], Haining Tian [a], Bruno Jousselme [c], Leif Hammarström [a], Tomas Edvinsson* [b]

[a] Department of Chemistry, Physical Chemistry, Ångström Laboratory, Uppsala University, Box 523, 751 20 Uppsala, Sweden

[b] Department of Materials Science and Engineering, Solid State Physics, Ångström laboratory, Uppsala University, Box 35, 751 03 Uppsala, Sweden

[c] Université Paris-Saclay, CEA, CNRS, NIMBE, LICSEN, 91191, Gif-sur-Yvette, France

* Tomas.Edvinsson@angstrom.uu.se

Table of Content

Synthesis 3

Characterization 3

SEM/EDX of as-synthesized $NiMoO_4$@Nif 5

Raman spectroscopy of as-synthesized $NiMoO_4$@Nif 14

ATR-FTIR of as $NiMoO_4$ nanostructures removed from as-synthesized Nif 17

XPS analysis of as-synthesized $NiMoO_4$@Nif 17

Electrochemistry 20

CV with normalization to geometric surface area of nickel foam 26

SEM after 500 cycles in 1.0 M KOH 27

XPS after 500 cycles in 1.0 M KOH 31

Selective Mo etched $NiMoO_4$@Nif-5 33

Time resolved *in situ* and *operando* Raman spectroscopy 34

ICP-OES after CPE 40

EDX after CPE 41

Bibliography 43

Synthesis

Nickel molybdate hydrate was synthesized in a common hydrothermal synthesis, inspired by Wang *et al*. and Zhang *et al*. [1,2]. 2.4 mmol (0.698 g) $Ni(NO_3)_2{\cdot}6H_2O$ was dissolved in 30 mL deionized water and stirred with a magnetic for approx. 20 minutes at 500 rpm. 2.4 mmol (0.582 g) $Na_2MoO_4{\cdot}2H_2O$ was also dissolved in 30 mL deionized water and slowly added after full dissolving to the prior solution under heavily stirring (500 rpm). The solution was stirred for 20 - 45 minutes. The pH value was measured as 6.8. In parallel the nickel foam (5 cm × 3 cm) was cleaned in an ultrasonic bath in EtOH to remove grease and other organic solvable dirt, followed by rinsing with deionized water and subsequent ultrasonic cleaning in 3 M HCl for 15 min each, to remove impurities and surface oxides. 3 M HCl solution was prepared by diluting 50 mL of 37 % (12 M) hydrochloride acid in 150 mL deionized water. The foam was then rinsed with deionized water and EtOH and blown dry under nitrogen stream. The cleaned Ni foam and the 0.04 M $Ni(NO_3)_2$ and 0.04 M Na_2MoO_4 solution was transferred into a Teflon lined stainless steel autoclave. The foam was slightly bend and placed against the wall. Previous synthesizes have shown, that close to the wall the most precipitation is taking place. The autoclave was sealed and directly heated up to 150 °C for 6 hours with 2 °C min^{-1} heating ramp. After the holding time it was allowed to cool down naturally. The yellow greenish $NiMoO_4{\cdot}nH_2O$ on nickel foam was ultrasonicated for 10 minutes in EtOH or 2-Propanol, to remove loose particles, followed by rinsing with the same solvent and dried over night at air. The sample is denoted $NiMoO_4$@Nif-2. For comparison, two more nickel molybdate hydrate on nickel foam was prepared by the same procedure, but with heating ramps of 0.5 °C min^{-1} and 5 °C min^{-1}, from here on called $NiMoO_4$@Nif-0.5 and $NiMoO_4$@Nif-5, respectively. We would like to clarify, that in all cases no annealing step is conducted and the hydrate structure of $NiMoO_4$ is still present, even if not indicated by the abbreviations.

Characterization

Ex situ Raman spectroscopy was conducted in the described setup under air with a 20 × magnification lens was used with a laser intensity of 5 %, which was adjusted with neutral density filters. Time resolved *in situ* and *operando* Raman spectra were collected in the same system with an L-shaped 10 × magnification lens, 50 % power intensity and 5 s acquisition time. The sample was in a quartz glass filled with 1.0 M KOH. There was no quiet time in between the measurements, however, since the program itself needed time for storing the data, a deviation of 2 - 3 s accumulated over 180 s. For *operando* measurements this means the very first and the very last acquisition are *in situ*. In the *in situ* at 0.95 V *vs* Reversible Hydrogen electrode (RHE) and all *operando* measurements the bias was applied after collecting the first spectra, to see if the bias caused any effects in the first seconds. For the *in situ* at 0.95 V *vs* RHE and the *operando* measurements a platinum counter electrode and a saturated

Ag/AgCl reference electrode was placed in the same cell. To facilitate the mounting and to assure a constant geometric adjustment of the electrode setup while moving the optical table for focusing, the quartz cuvette and the wires and electrodes were fixed with a Teflon cap and a 3D printed cuvette holder on the optical table of the spectroscope. The power intensity on the sample was measured for the most common acquisition conditions with a PM 160 optical power meter. The 20 × magnification lens with a laser intensity of 5 % showed a laser power of 5.53 mW on the sample. The samples measured with the L-shaped lens with 10 × magnification, used for *in situ* and *operando* Raman spectroscopy experienced a laser power between 22.5 mW (measured before the quartz glass) and 18.4 mW (behind the quartz glass with electrolyte). However, since we could not determine the area the laser beam is exciting, we cannot quantify the power density on the samples.

Complementary attenuated total reflection Fourier-transformed infrared spectroscopy (ATR-FTIR) analysis was acquired with a PerkinElmer Spectrum One with a diamond/ZnSe crystal and the corresponding Spectrum (V.5.0.2) software. ATR-FTIR was the only technique for which $NiMoO_4 \cdot nH_2O$ powder was used. We obtained it by extensive ultrasonication of the $NiMoO_4$@Nif in EtOH or 2-propanol (1 h). For all other characterization techniques $NiMoO_4$@Nif was investigated.

Electrochemical measurements were done in the system described before. The potential of the reference electrode was checked prior to use. The reference electrode was prepared according to literature.[3] The three-compartment cell was stored overnight in 3 M HCl to remove impurities, followed by subsequent intensive rinsing with DI and acetone. As electrolyte a 1.0 M KOH was prepared from dissolving 14.03 g of KOH pellets in 250 mL ultrapure water. We confirmed pH 14 with pH paper. For each electrochemical measurement fresh electrolyte was used. The double layer capacitance (C_{dl}) of the WE was detected by cyclic voltammetry (CV) between 0.9 V – 1.0 V *vs* RHE with scan rates of 150, 120, 100, 80, 60, 40, 20, 10 and 5 mV s^{-1} for the bare nickel foam and $NiMoO_4$@Nif. Since the Ni^{III}/Ni^{II} reduction is sluggish, the reduction current interfered with the capacitive current for the ECSA measurement after catalysis, leading to a drift of the CVs for measurement. In order to decrease the effect of this interference, only the scan rates from 150 mV s^{-1} to 40 mV s^{-1} were used, since they showed the most stable behavior (**SI Figure 19**). All scans were not ohmic drop (iR) compensated. For each scan rate we took two cycles and used the second one for determining the difference in current density of forward and backward scan at 0.95 V *vs* RHE. This difference was plotted *versus* the scan rate. The slope of the linear fit of those data points is the double of the double layer capacitance and proportional to the electrochemical surface area (ECSA), following equation (**SI 1**).

$$C_{dl} = ECSA \cdot C_s \quad \textbf{(SI 1)}$$

With ECSA being the electrochemical surface area, C_{dl} the double layer capacitance and C_s the specific capacitance of the material. Under the assumption of a constant specific capacitance the C_{dl} can be directly can compared to the ECSA. However, we here only report the values of the double layer capacitance.

For inductive coupled plasma – optical emission spectrometry (ICP-OES) the blank and washing solution for all experiments was H_2O (ASTM Type I). The standard solutions of 100 mg·L^{-1}, 10 mg·L^{-1} and 1 mg·L^{-1} Mo in H_2O where used with the corresponding blank solution. For achieving the desired dilution, the supplied 1000 µg·mL^{-1} Mo in H_2O standard solutions was diluted 10, 100 and 1000 times, respectively. As reagent blank the 1.0 M KOH of the as prepared electrolyte was used. The electrolyte to analyze was filtered prior to analysis with a 0.2 µm PTFE filter or 0.2 µm Nylon filter to remove possible nanoparticles. The molybdenum signal was detected at 202.031 nm. All blanks, standard solutions, reagent blanks and samples were analyzed with three replicates.

For measuring the pH value of the hydrothermal synthesis solution we used a TES 1380 pH meter.

SEM/EDX of as-synthesized $NiMoO_4$@Nif

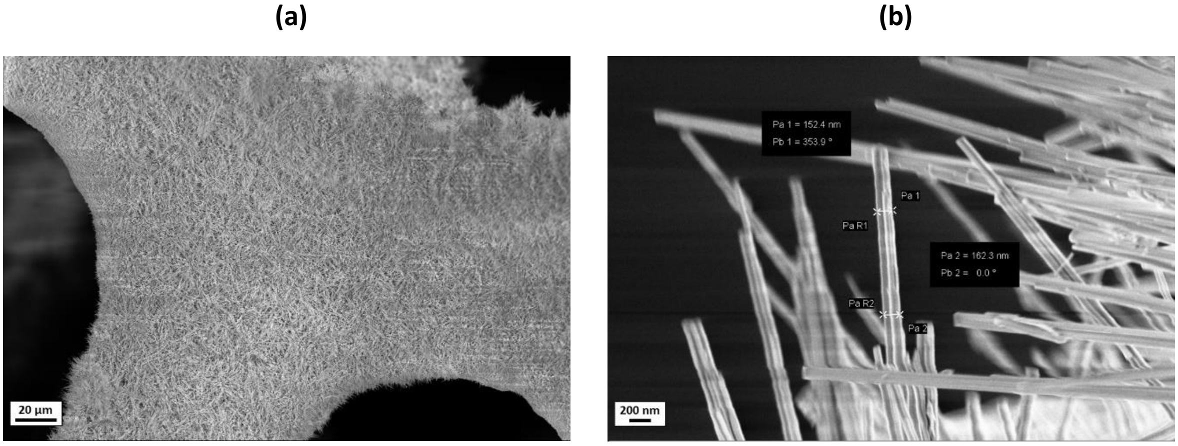

SI Figure 1: SEM analysis of $NiMoO_4$@Nif-2. **(a)** – **(b)** SEM secondary electron imaging at different magnifications, showing a homogeneous coated foam with only nanorods being visible.

For $NiMoO_4$@Nif-2 only nanorods were observed in the scanning electron microscopy (SEM) images.

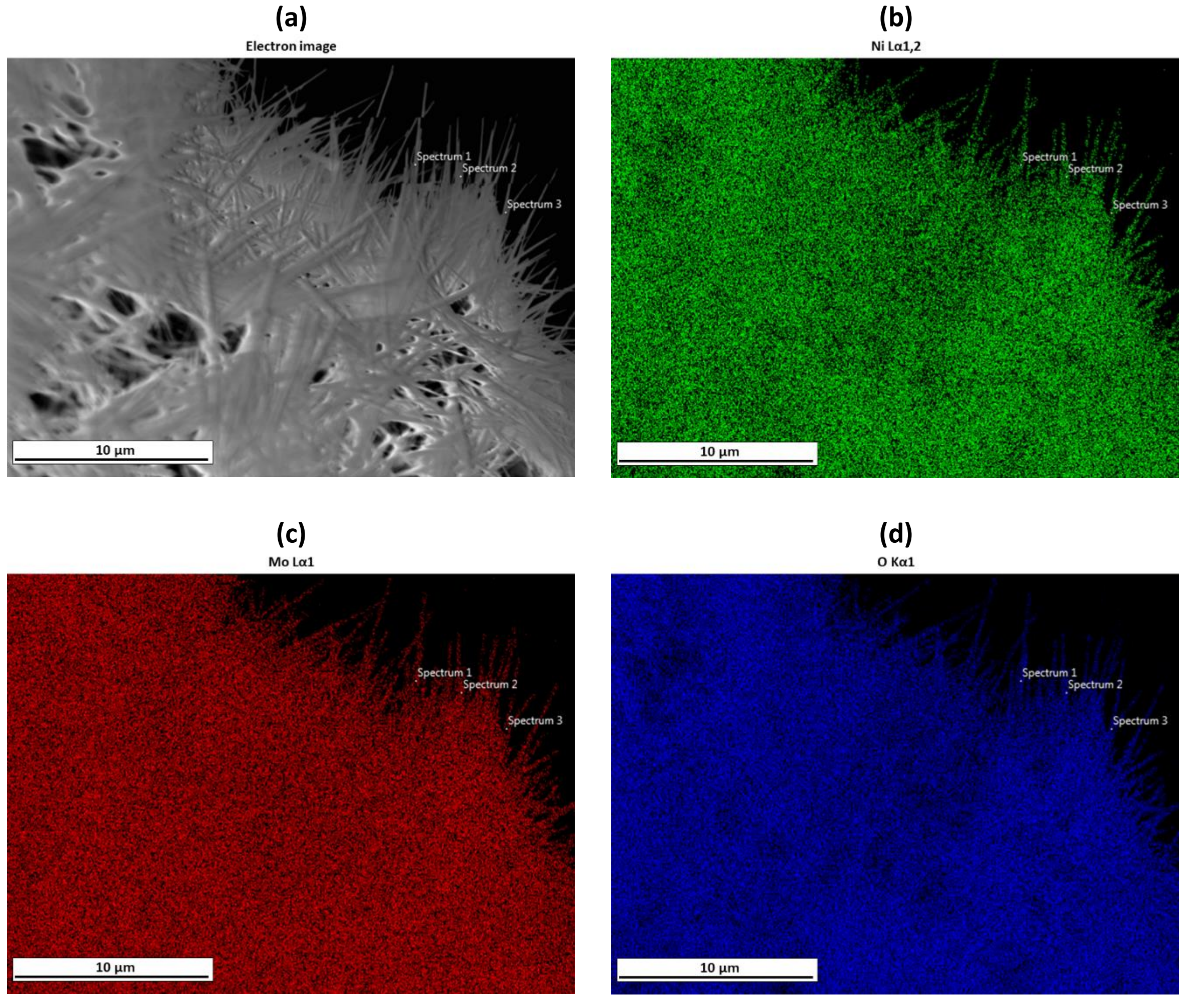

SI Figure 2: EDX mapping of Ni, Mo and O in NiMoO4@Nif-2. Spectrum 1 – 3 indicate the spots for point ID analysis, which are shown in **SI Figure 3**. **(a)** Secondary electron image. **(b)** Nickel distribution. **(c)** Molybdenum distribution. **(d)** Oxygen distribution.

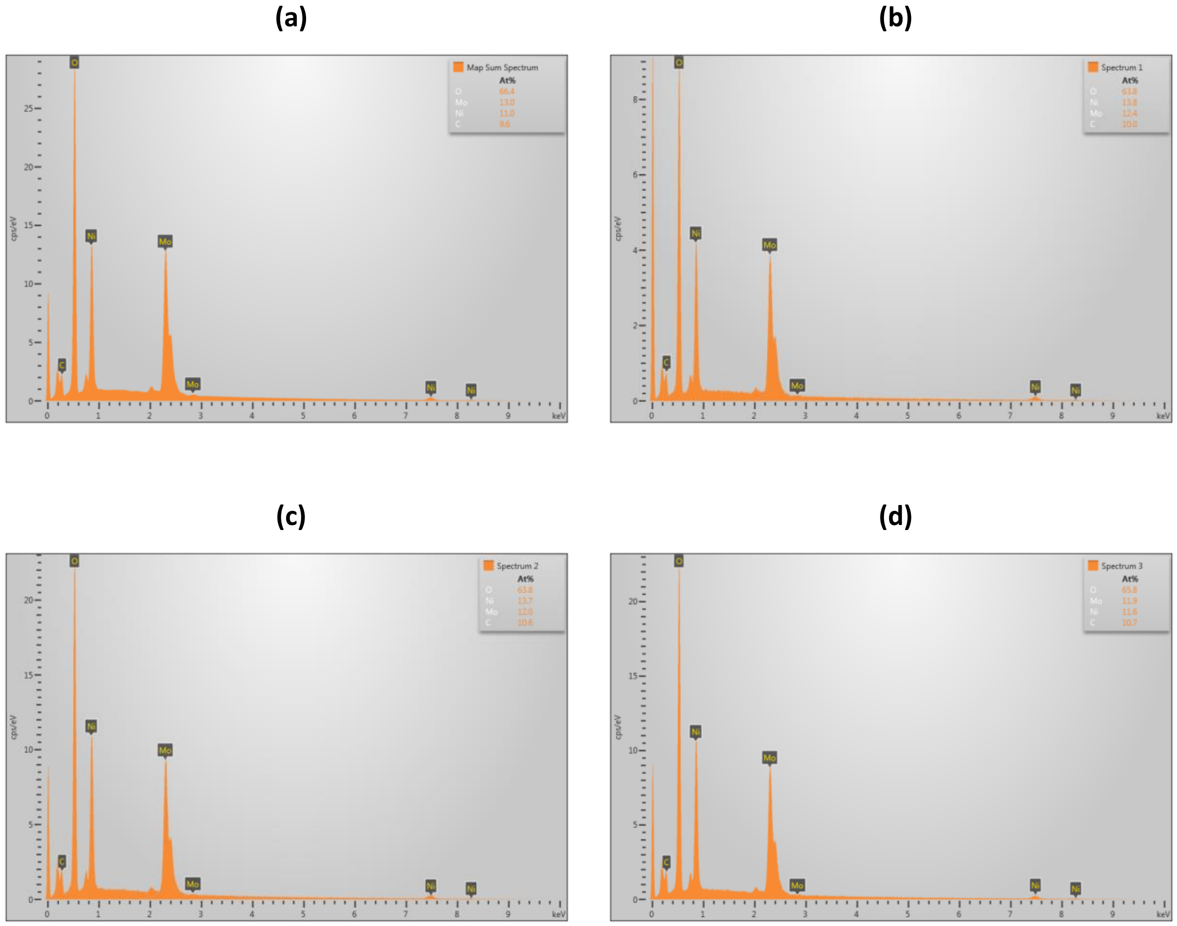

SI Figure 3: EDX analysis of $NiMoO_4$@Nif-2. **(a)** Spectrum of the EDX mapping in **SI Figure 2**. **(b)** – **(d)** EDX single ID spectra of spots 1 – 3 as indicated in **SI Figure 2**.

The energy dispersive X-ray (EDX) spectra of the mapping and the singe spot IDs shows a roughly equal amount of nickel and molybdenum in the nanorod structures, partly even with a slightly higher amount of molybdenum.

Scanning electron microscopy (SEM) images of $NiMoO_4$@Nif-0.5 shows a majorly nanorod structures with some dispersed nanoflowers, primarily in clusters in regions without nickel foam.

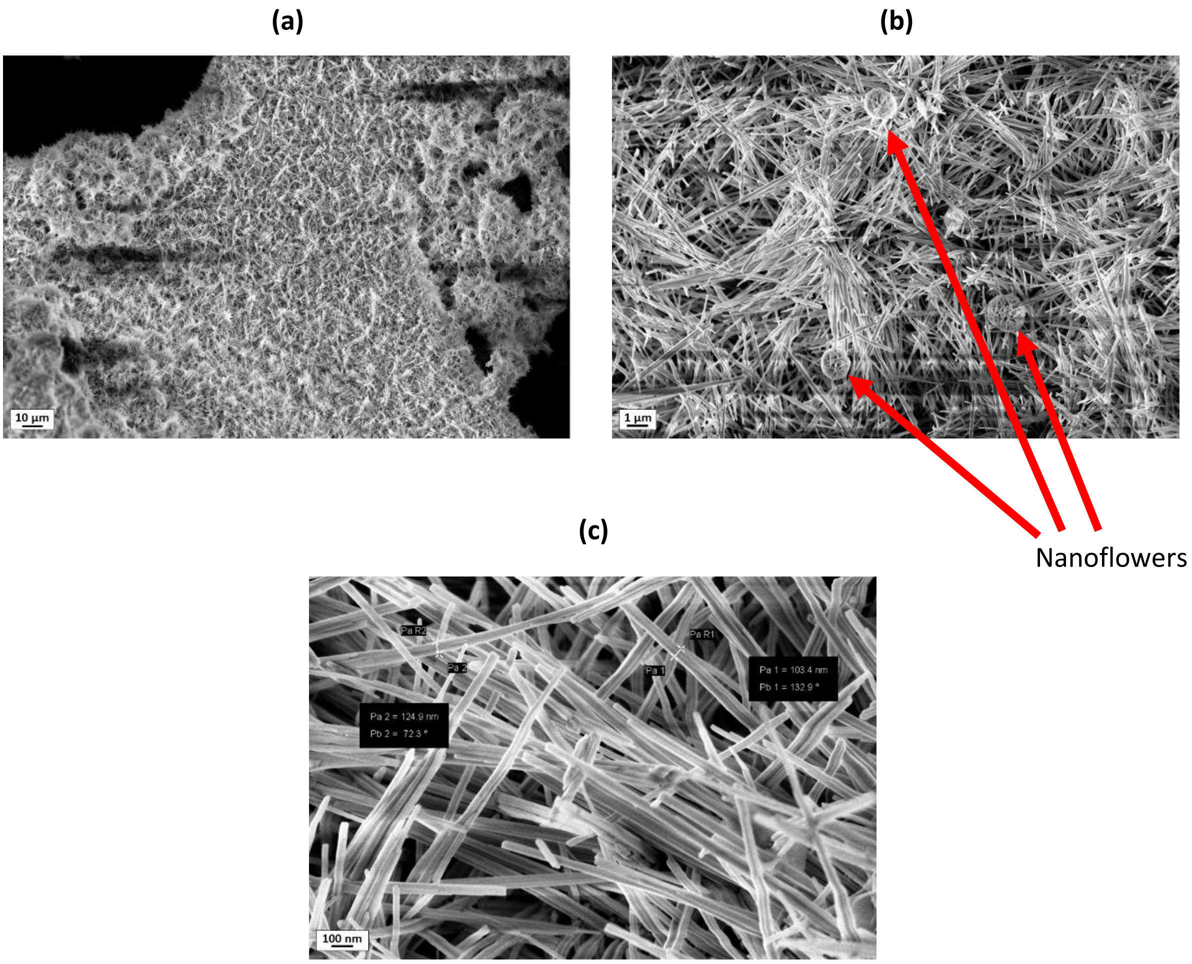

SI Figure 4: SEM secondary electron images of $NiMoO_4$@Nif-0.5 with **(a)** – **(c)** different magnifications.

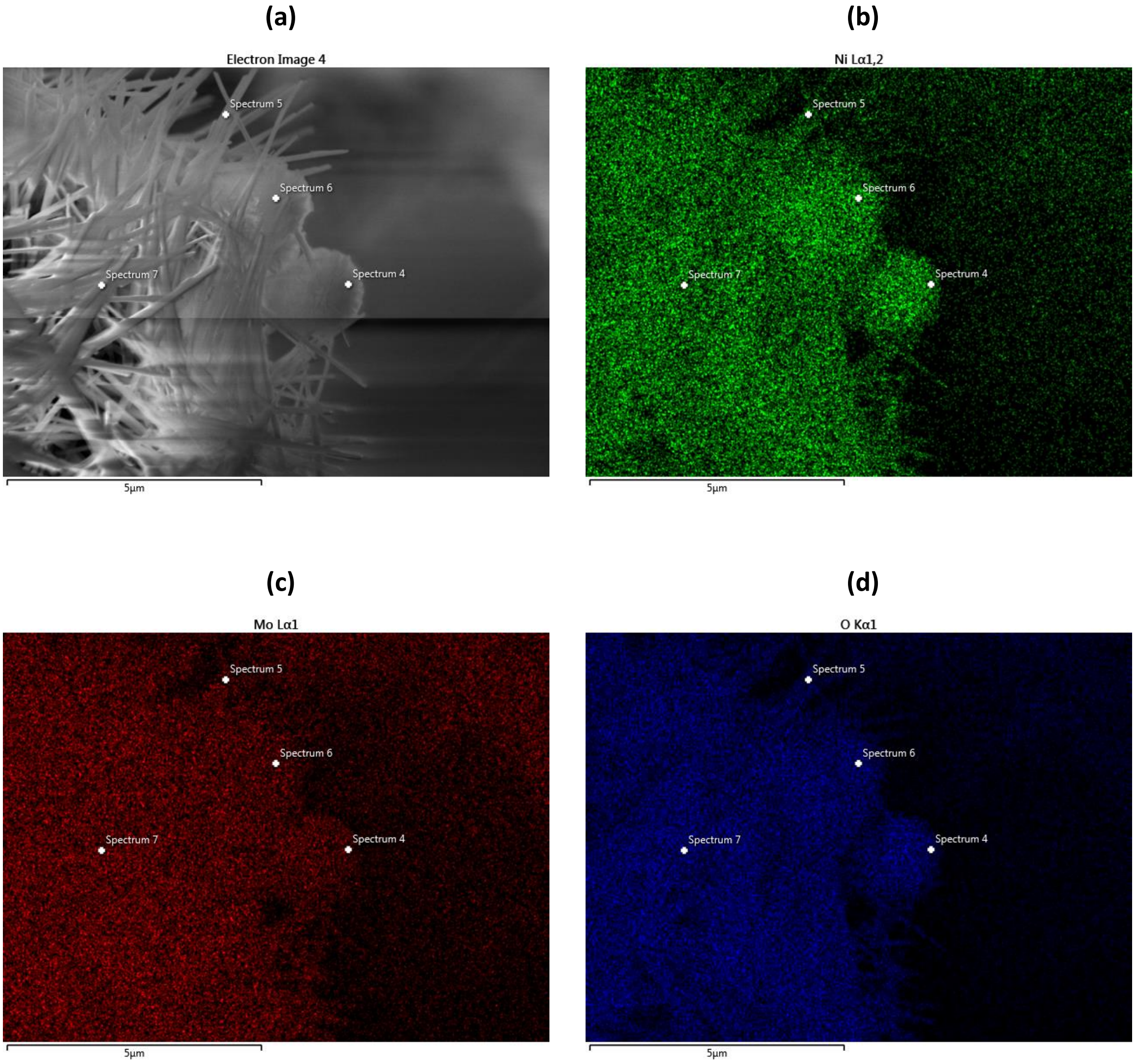

SI Figure 5: EDX mapping of Ni, Mo and O in $NiMoO_4$@Nif-0.5. Spectrum 4 – 7 indicate the spots for point ID analysis, which are shown in **SI Figure 6**. **(a)** Secondary electron image. **(b)** Nickel distribution. **(c)** Molybdenum distribution. **(d)** Oxygen distribution.

EDX mapping of the nanostructures of $NiMoO_4$@Nif-0.5 show a homogeneous dispersion of nickel, molybdenum and oxygen.

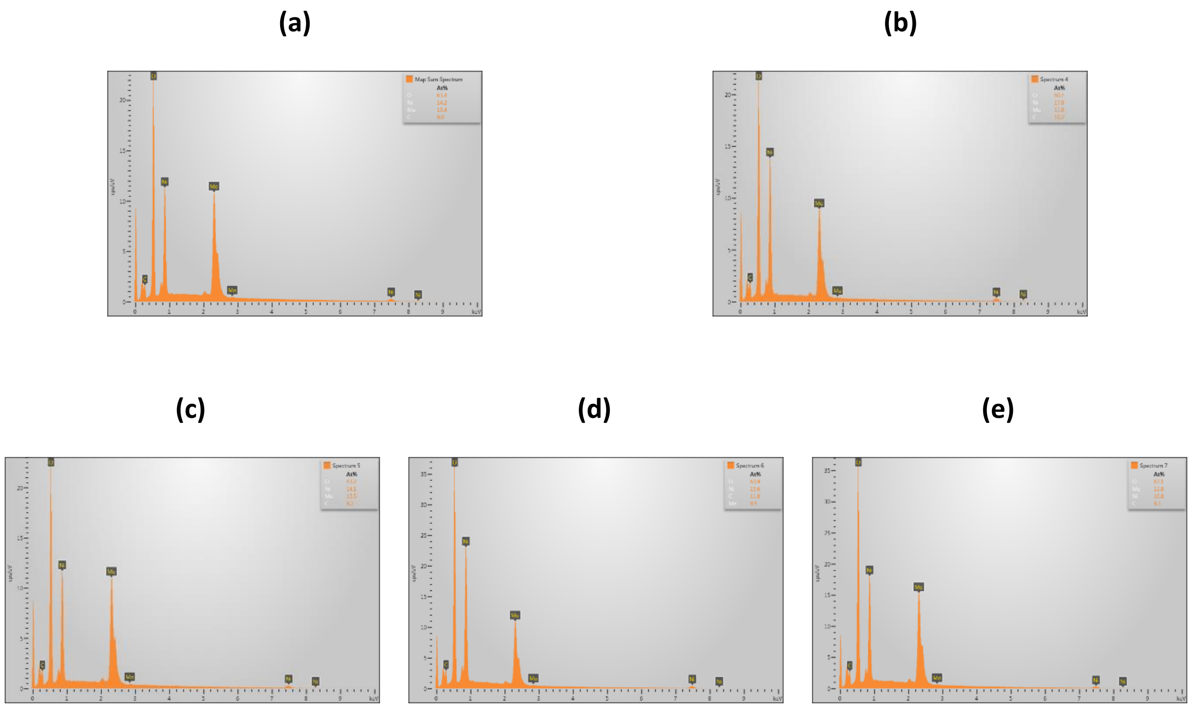

SI Figure 6: EDX analysis of $NiMoO_4$@Nif-0.5. **(a)** EDX spectrum of the mapping of **SI Figure 5**. **(b)** – **(e)** EDX single ID spectra of spots 4 – 7 as indicated in **SI Figure 5**.

The map spectrum shows an overall similar amount of nickel and molybdenum with a slightly larger atomic percentage of the former. The amount of oxygen is a bit more than four times larger. For the spectra 4 – 7 the overall amount ratio of nickel to molybdenum is higher for the flowerlike structure, whereas the amount of molybdenum increases at spots with nanorods.

(a) **(b)**

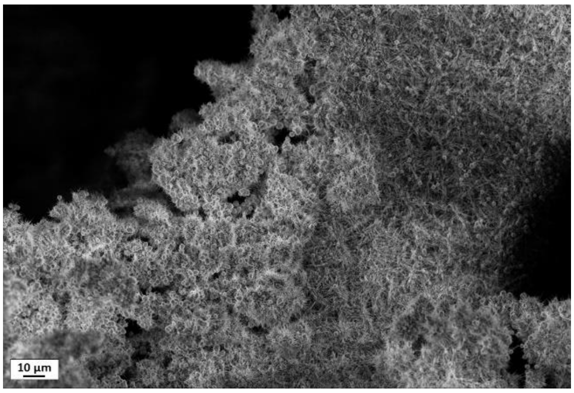

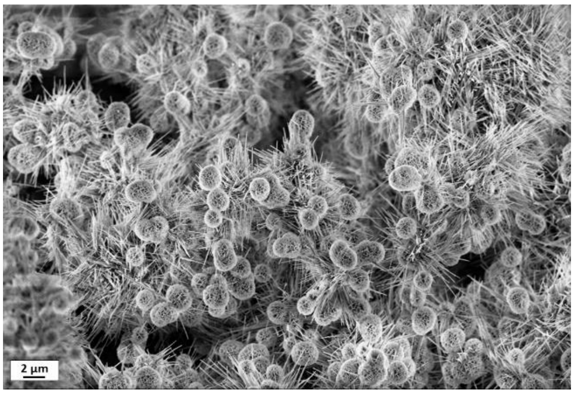

(c)

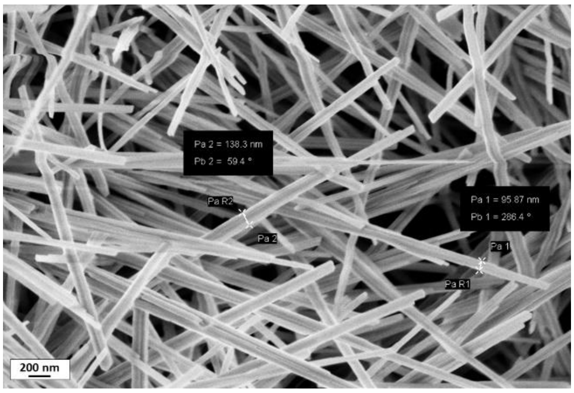

SI Figure 7: SEM secondary electron images of $NiMoO_4$@Nif-5 with **(a)** – **(c)** different magnifications.

The SEM investigation of $NiMoO_4$@Nif-5 has shown of nanorods, but also the highest amount of nanoflowers among all samples. Those nanoflowers seem to preferably cluster in distance to the nickel foam.

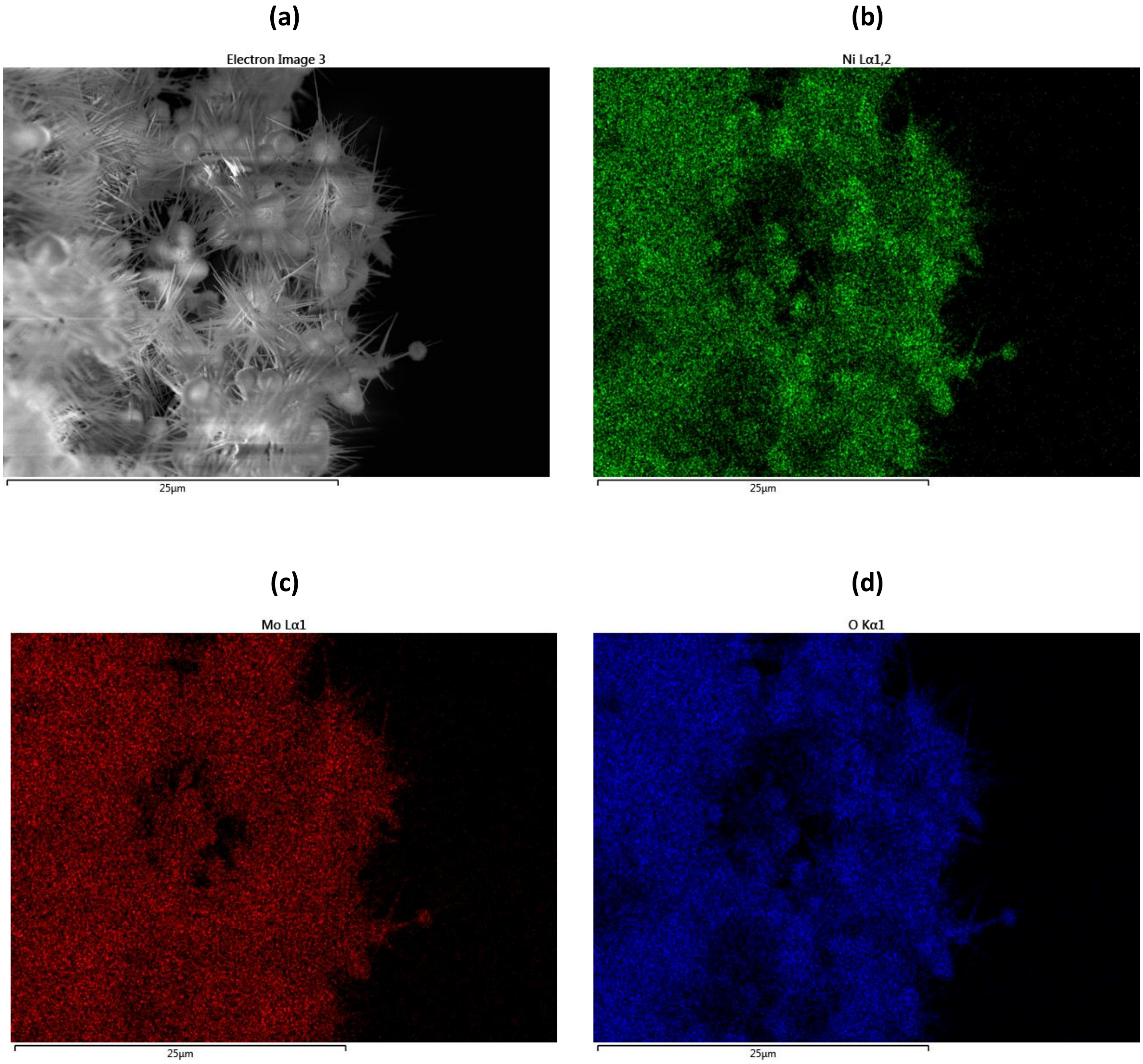

SI Figure 8: EDX mapping of Ni, Mo and O in $NiMoO_4$@Nif-5. **(a)** Secondary electron image of the mapping area. **(b)** Nickel distribution. **(c)** Molybdenum distribution. **(d)** Oxygen distribution.

As for the other samples also for $NiMoO_4$@Nif-5 the EDX analysis reveals a homogeneous distribution of nickel, molybdenum and oxygen in the sample.

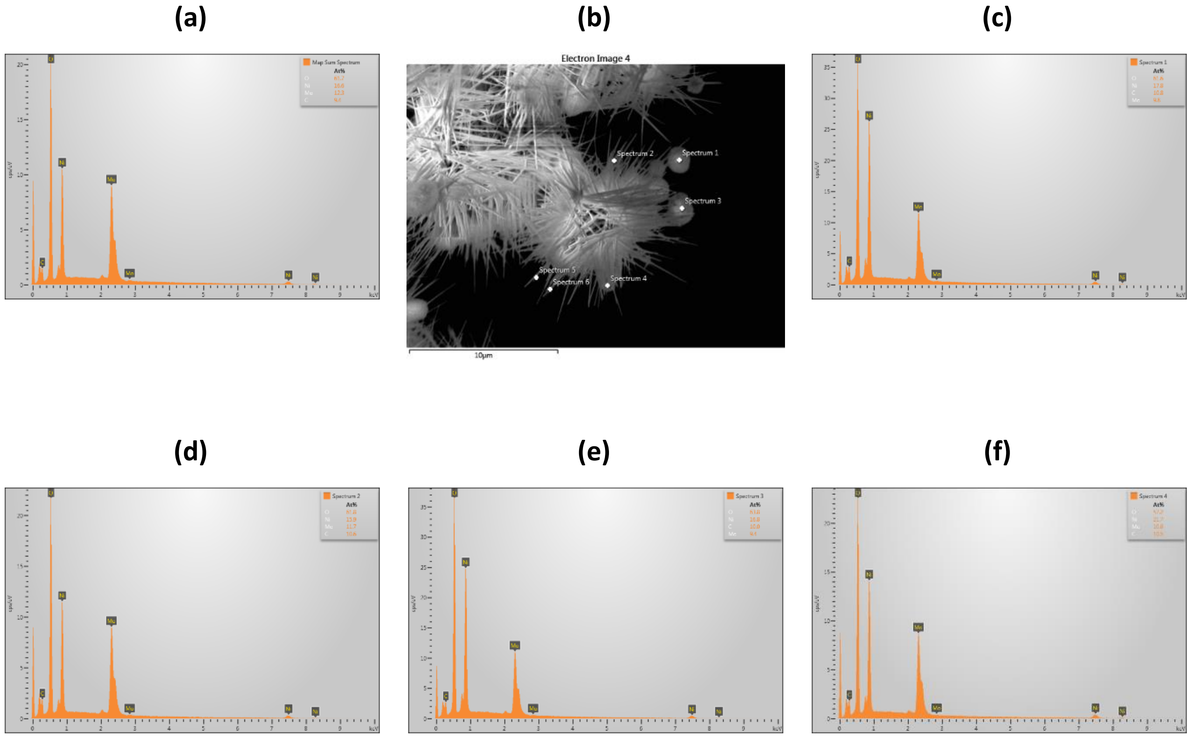

SI Figure 9: EDX analysis of $NiMoO_4$@Nif-5. **(a)** The spectrum of the mapping in **SI Figure 8**. **(b)** Secondary electron image with indicating the EDX point ID spots for the spectra in **(c)** – **(f)**.

In general, the EDX spectra of $NiMoO_4$@Nif-5 again shows the reported trend with having a higher amount of nickel compared to molybdenum. This is especially visible in the point ID spectra of the nanoflower in **(c)** and **(e)** compared with the spectrum with the nanorod in **(d)**. However, in all spectra the oxygen percentage is lower than in the other samples and don't reach four times the amount of nickel. Due to charging effect and drift of the image, no trustworthy EDX analysis could be conducted for spectrum 5 and 6.

The elemental concentration and the Ni:Mo ratio is displayed and calculated in the table below.

SI Table 1: Elemental composition and Ni:Mo atomic ratio detected by EDX.

Sample	Sum/Spot	Nanostructure	c in at.%			Ni:Mo ratio
			Ni	Mo	O	
$NiMoO_4$@Nif-2	Sum		11	13	66.4	0.85
	Spot 1	Nanorod	13.8	12.4	63.8	1.11
	Spot 2	Nanorod	13.7	12	63.8	1.14
	Spot 3	Nanorod	11.6	11.9	65.8	0.97

$NiMoO_4$@Nif-0.5	Sum		14.2	13.4	63.4	1.06
	Spot 4	Nanoflower	17.8	11.6	60.4	1.53
	Spot 5	Nanorod	14.1	13.5	63.2	1.04
	Spot 6	Nanoflower/ Nanorod	15.6	9.5	63.9	1.64
	Spot 7	Nanorod	10.8	12.8	67.1	0.84
$NiMoO_4$@Nif-5	Sum		16.6	12.3	61.7	1.35
	Spot 1	Nanoflower	17.8	9.8	61.6	1.82
	Spot 2	Nanorod	15.9	11.7	61.8	1.36
	Spot 3	Nanoflower	16.8	9.4	63.8	1.79
	Spot 4	Nanoflower	21.7	10.6	57.2	2.05

Raman spectroscopy of as-synthesized $NiMoO_4$@Nif

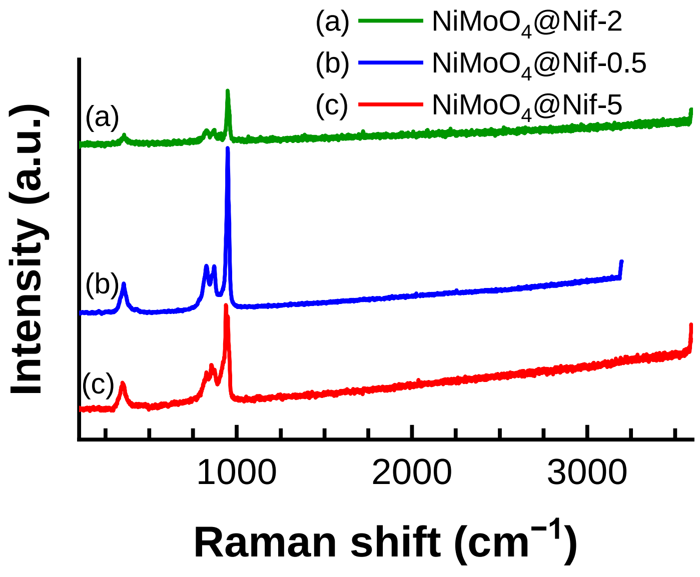

SI Figure 10: Extensive scans up to 3600 cm^{-1} with different laser intensities for $NiMoO_4$@Nif-2 (0.1 %), $NiMoO_4$@Nif-0.5 (5 %) and $NiMoO_4$@Nif-5 (1 %).

For better comparison the intensities were adjusted by multiplying $NiMoO_4$@Nif-2 with factor 5 $NiMoO_4$@Nif-0.5 with factor 0.7 and $NiMoO_4$@Nif-5 with factor 3.

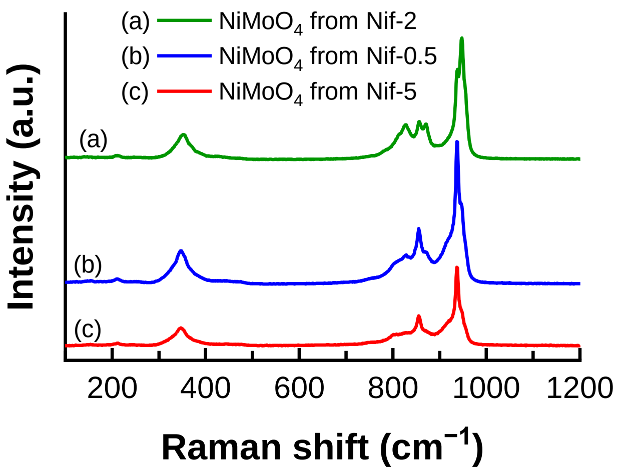

SI Figure 11: Raman spectra of the $NiMoO_4$ powders for ATR-FTIR. Taken with 20 × magnification and a laser intensity of 5 %.

The $NiMoO_4{\cdot}H_2O$ powder removed from the foam by extensively ultrasonication shows the same peaks as on the foam, only with a higher amount off flower-$NiMoO_4$. The powder from $NiMoO_4$@Nif-2 shows the highest amount of the rod-$NiMoO_4$ among all samples.

(a) (b)

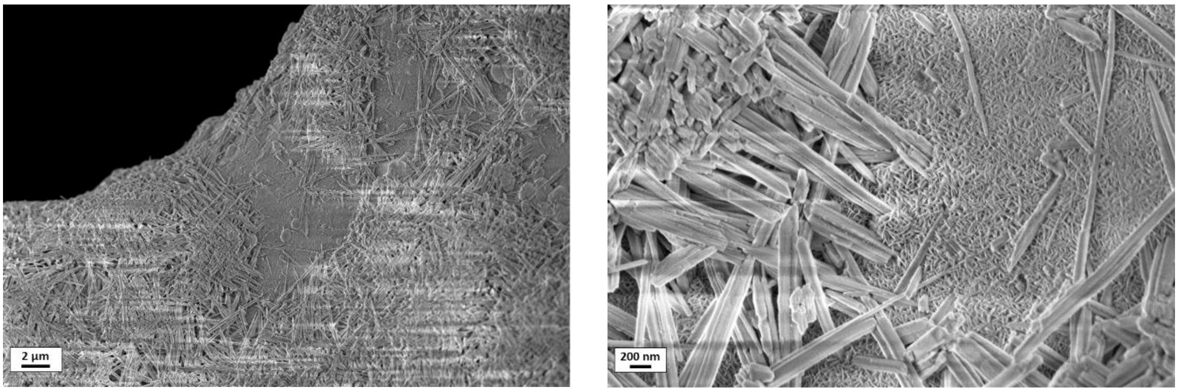

(c)

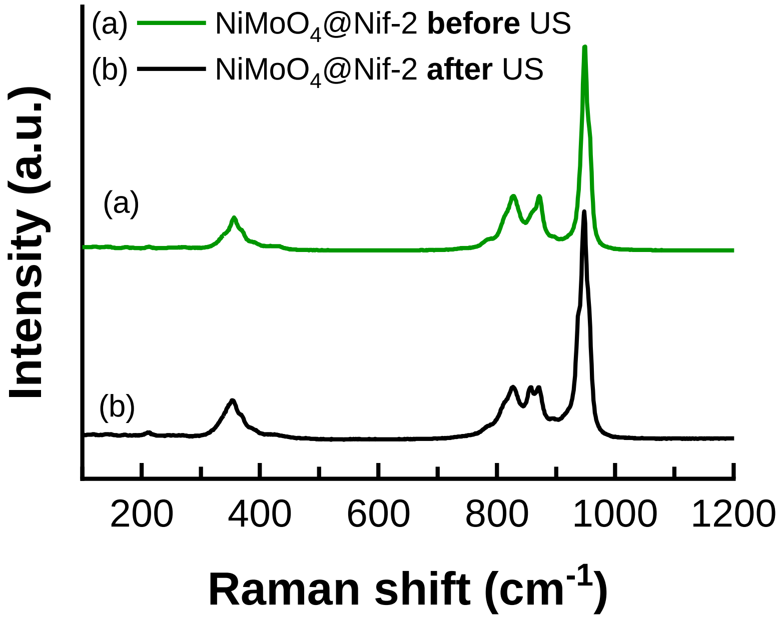

SI Figure 12: Investigating the effect of extensive ultrasonication on $NiMoO_4$@Nif-2. **(a)**-**(b)** SEM secondary electron images with different magnifications. **(c)** Raman spectroscopy of the sample before extensive ultrasonication (taken with 5 % laser intensity) and after ultrasonication (taken with 10 % laser intensity).

SEM reveals the removal of nanorods and the presence of nanoflower flake structure completely coating the nickel foam below the nanorods. Raman spectroscopy of the foam before and after extensive ultrasonication shows a change of intensities between the two molybdate species, with an increased signal from the flower structure after the ultrasonication.

ATR-FTIR of as $NiMoO_4$ nanostructures removed from as-synthesized Nif

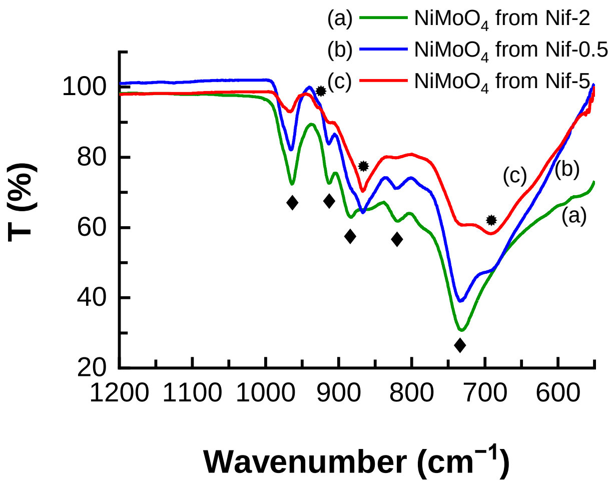

SI Figure 13: ATR-FTIR of the $NiMoO_4$ nanostructures, removed from the corresponding foam by extensive ultrasonication in alcohol.

ATR-FTIR showed as Raman Spectroscopy vibration signals of both rod-$NiMoO_4$ and flower-$NiMoO_4$. The powders are exactly the same as investigated with Raman in **SI Figure 11**, indicating a higher intensity of flower-$NiMoO_4$ vibrations as it would have been detected directly on the foam.

XPS analysis of as-synthesized $NiMoO_4$@Nif

X-ray photoelectron spectroscopy (XPS) was utilized to detected the chemical elements and the chemical composition of those elements on the surface of the as synthesized foams. The area of the orbitals was taken from the high-resolution elemental analysis, analyzed with CasaXPS 2.3.24, except for $NiMoO_4$@Nif-0.5, in which the survey scan was used for quantification. The RSF library of the commercial MultiPak 9.8.0.19 software was used for quantification. The slight deviation in binding energies for the same element among the different samples is due to the low adventitious carbon signal. Hence the maximum of the was not always unambiguous detectable, which results in a slightly different shifts in binding energies during the correction.

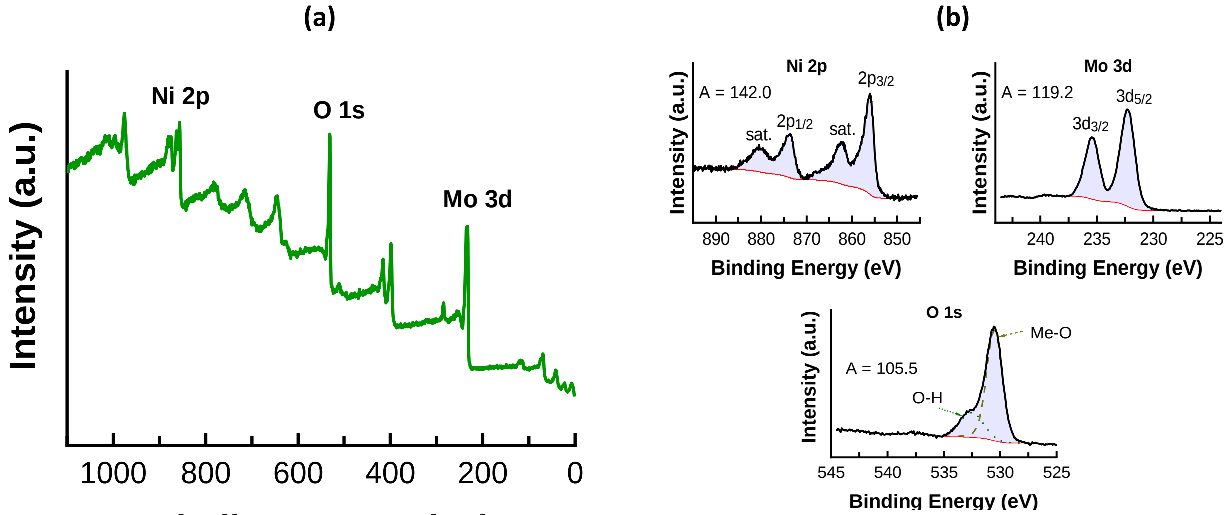

SI Figure 14: XPS spectra of $NiMoO_4$@Nif-2. **(a)** Survey spectrum. **(b)** High resolution elemental analysis of Ni 2p, Mo 3d and O 1s with the area used for the atomic ratio calculation.

The binding energies of $NiMoO_4$@Nif-2 are described in the publication. The atomic concentrations are illustrated in **SI Table 2**.

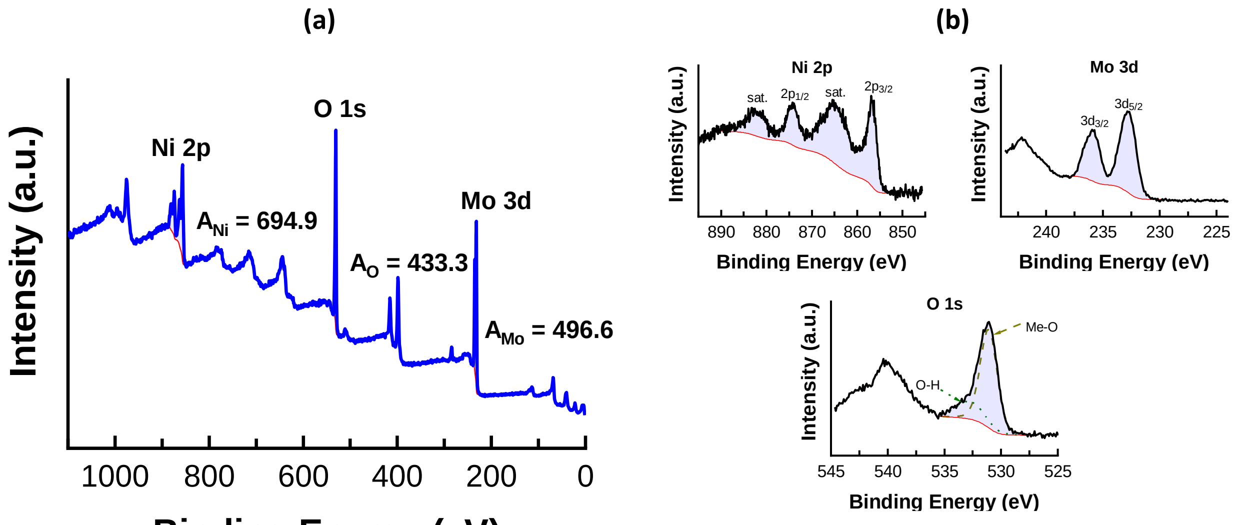

SI Figure 15: XPS analysis of $NiMoO_4$@Nif-0.5. **(a)** Survey scan and area of regions used for the calculation of the elemental ratio. **(b)** High resolution elemental analysis of nickel, molybdenum and oxygen.

For $NiMoO_4$@Nif-0.5 the elemental analysis was difficult to interpret. Therefore, the elemental concentration was taken from the areas in survey spectrum. The oxygen peak in **(b)** shows a combination of hydroxide with a binding energy of 532.7 eV and metal oxide at 531.1 eV. Nickel $2p_{3/2}$

and $2p_{1/2}$ was detected at 856.6 eV and 874.1 eV. Molybdenum $3d_{5/2}$ and $3d_{3/2}$ appear at 232.8 eV and 236.0 eV.

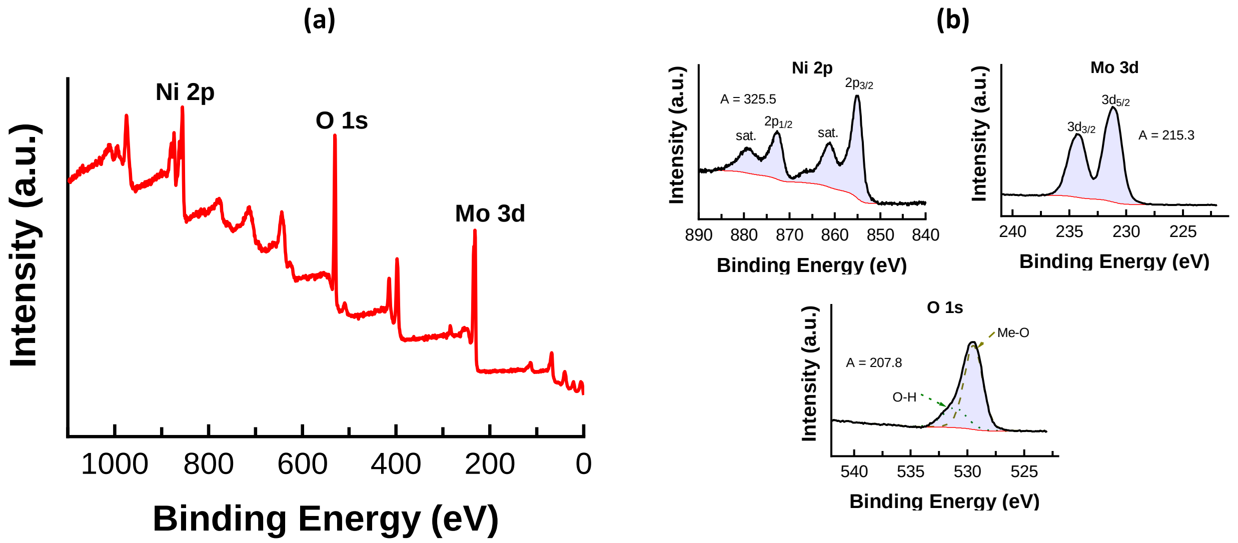

SI Figure 16: XPS analysis of $NiMoO_4$@Nif-5. **(a)** Survey spectrum. **(b)** High-resolution elemental analysis of nickel, molybdenum and oxygen with the area for calculating the elemental ratio.

The XPS spectrum of $NiMoO_4$@Nif-5 also shows an increased nickel content. As for the others, also the high-resolution analysis of O 1s can be divided in a hydroxide part at 531.2 eV and metal oxide part at 529.5 eV. Nickel $2p_{3/2}$ was detected at 855.1 eV and $2p_{1/2}$ at 872.6 eV. As already mentioned, Mo $3d_{5/2}$ and $3d_{3/2}$ appears at 231.1 eV and 234.3 eV, respectively.

SI Table 2: Calculation of elemental ratio of $NiMoO_4$@Nif.

	Orbital	Area	RSF	Corrected RSF	Area/ corr. RSF	at.%
$NiMoO_4$@Nif-2	Ni 2p	142	3.702	4.05739	35.00	16.8
	Mo 3d	119.2	3.544	4.03071	29.57	14.2
	O 1s	105.5	0.733	0.733	143.93	69.0
$NiMoO_4$@Nif-0.5	Ni 2p	694.9	3.702	4.05739	171.27	19.3
	Mo 3d	496.6	3.544	4.03071	123.20	13.9
	O 1s	433.3	0.733	0.733	591.13	66.8
$NiMoO_4$@Nif-5	Ni 2p	325.5	3.702	4.05739	80.22	19.2
	Mo 3d	215.3	3.544	4.03071	53.41	12.8
	O 1s	207.8	0.733	0.733	283.49	68.0

Electrochemistry

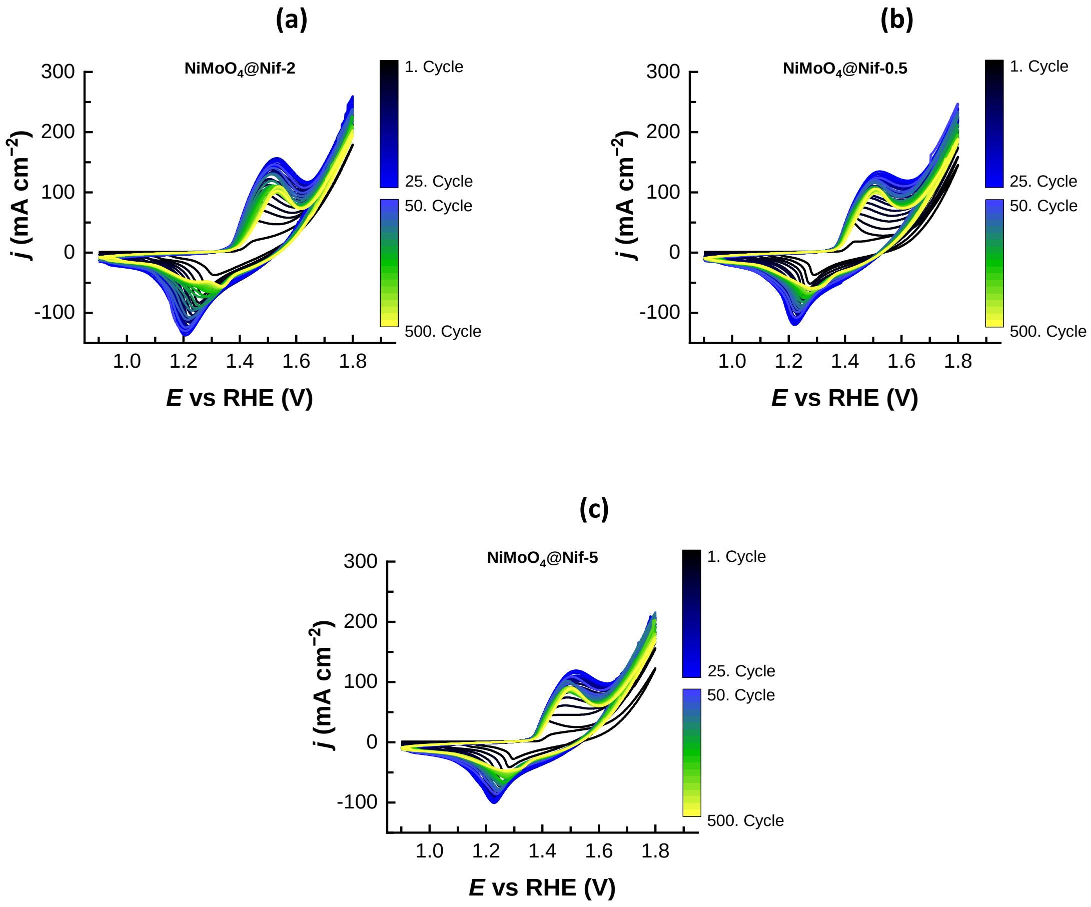

SI Figure 17: CV with 500 cycles in 1.0 M KOH with 10 mVs^{-1} and no iR compensation. Illustrated are cycles 1 – 25 and every 25th after. **(a)** NiMoO$_4$@Nif-2. **(b)** NiMoO$_4$@Nif-0.5. **(c)** NiMoO$_4$@Nif-5.

The surface coverage Γ with nickel is calculated with

$$\Gamma = \frac{Q_{\mathrm{Ni^{III}}/\mathrm{Ni^{II}}}}{F \cdot A} \qquad \textbf{(SI 2)}$$

With $Q_{\mathrm{Ni^{III}}/\mathrm{Ni^{II}}}$ being the charge consumed for the oxidation from Ni^{II} to Ni^{III} in the cyclic voltammetry (CV), F being Faradays constant (96 485.33 C mol^{-1}) and A being the surface area of the working electrode (0.25 cm^2 for NiMoO$_4$@Nif-0.5 and NiMoO$_4$@Nif-5, 0.24 cm^2 for NiMoO$_4$@Nif-2). The charge consumed for the oxidation was detected by analyzing the area under the oxidation peak in the CHI software. We took the average of three replicates for the first cycle and the cycle with the highest oxidation peak current.

SI Table 3: Surface coverage by charge analysis of the CVs of $NiMoO_4$@Nif-2 in **SI Figure 17 (a)**.

$NiMoO_4$@Nif-2		1. Cycle	20. Cycle
$Q_{\mathrm{Ni^{III}/Ni^{II}}}$	Measurement 1	0.01473 C	0.31230 C
	Measurement 2	0.01401 C	0.31130 C
	Measurement 3	0.01439 C	0.31390 C
	Average	0.01438 C	0.31250 C
Γ		0.6208 µmol cm^{-2}	13.50 µmol cm^{-2}

SI Table 4: Surface coverage by charge analysis of the CVs of $NiMoO_4$@Nif-0.5 in **SI Figure 17 (b)**.

$NiMoO_4$@Nif-0.5		1. Cycle	25. Cycle
$Q_{\mathrm{Ni^{III}/Ni^{II}}}$	Measurement 1	0.01269 C	0.34550 C
	Measurement 2	0.01297 C	0.34070 C
	Measurement 3	0.01296 C	0.32610 C
	Average	0.01287 C	0.33743 C
Γ		0.5337 µmol cm^{-2}	13.99 µmol cm^{-2}

SI Table 5: Surface coverage by charge analysis of the CVs of $NiMoO_4$@Nif-5 in **SI Figure 17 (c)**.

$NiMoO_4$@Nif-5		1. Cycle	23. Cycle
$Q_{\mathrm{Ni^{III}/Ni^{II}}}$	Measurement 1	0.00825 C	0.29060 C
	Measurement 2	0.00970 C	0.28590 C
	Measurement 3	0.00990 C	0.28340 C
	Average	0.00928 C	0.28663 C
Γ		0.3848 µmol cm^{-2}	11.88 µmol cm^{-2}

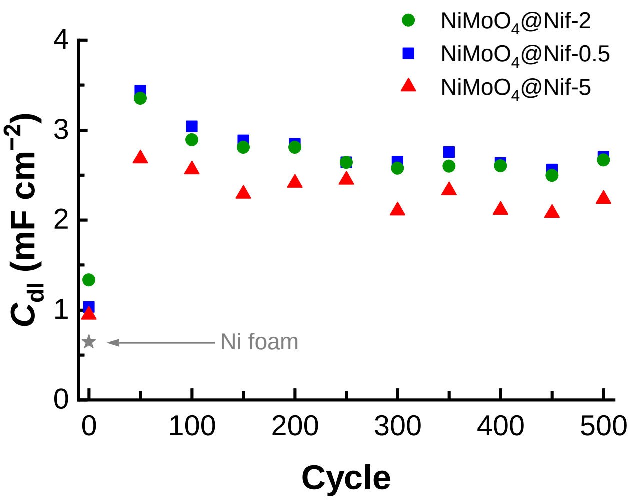

SI Figure 18: Double layer capacitance before and after every 50 cycles for $NiMoO_4$@Nif-2 (green circles), $NiMoO_4$@Nif-0.5 (blue cubes) and $NiMoO_4$@Nif-5 (red triangles)

Double layer capacitance (C_{dl}) detected by analysis of the non-faradaic current density at 0.95 V *vs* RHE in dependence of the scan rate. The ECSA is proportional to the C_{dl}, as shown in equation **SI 1**. Due to the sluggish reduction of Ni^{III} to Ni^{II} it contributes faradaic current even at very low potentials. Hence it is challenging to determine the C_{dl} without its simultaneous influence on the current. After determining the C_{dl} with different ranges of scan rate, we detect only slight changes in the value of the C_{dl} (**SI Figure 19 (b)**). From the retained trend in a clearly increased C_{dl}, we can conclude that the double layer capacitance markedly increased due to the CV compared to the as synthesized electrode. However, an exact value for the C_{dl} for each condition would be uncertain.

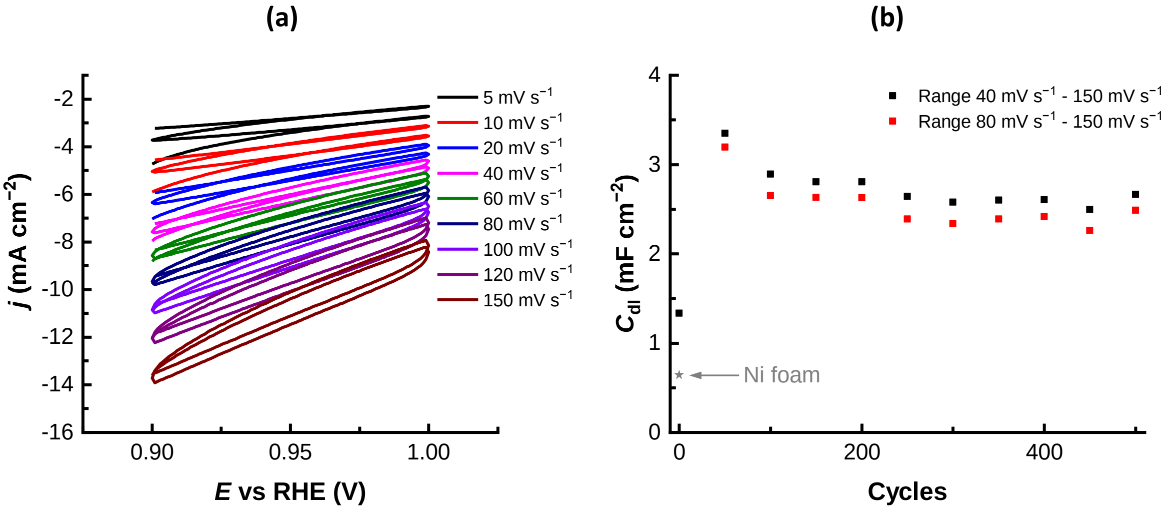

SI Figure 19: Influence of Ni^{III}/Ni^{II} reduction on the determination of C_{dl}. **(a)** The faradaic current causes a shift of the CVs, which are used for determining the C_{dl}. **(b)** Influence of changing the range of scan rates used for detecting the C_{dl}. Including lower scan rates results in a higher C_{dl} detected.

When detecting the C_{dl} by using different scan rates, the CVs of lower scan rates show more drift because more charge is consumed during the acquisition time in the simultaneously reduction of Ni^{III} to Ni^{II}. Excluding low scan rates minimizes this deviation on the C_{dl}. Including the scan CVs taken with a scan rate of 40 mV s^{-1} and 60 mV s^{-1} results in an overestimated C_{dl}, however, since the difference to C_{dl} detected excluding them is only minor, we not consider this as crucial.

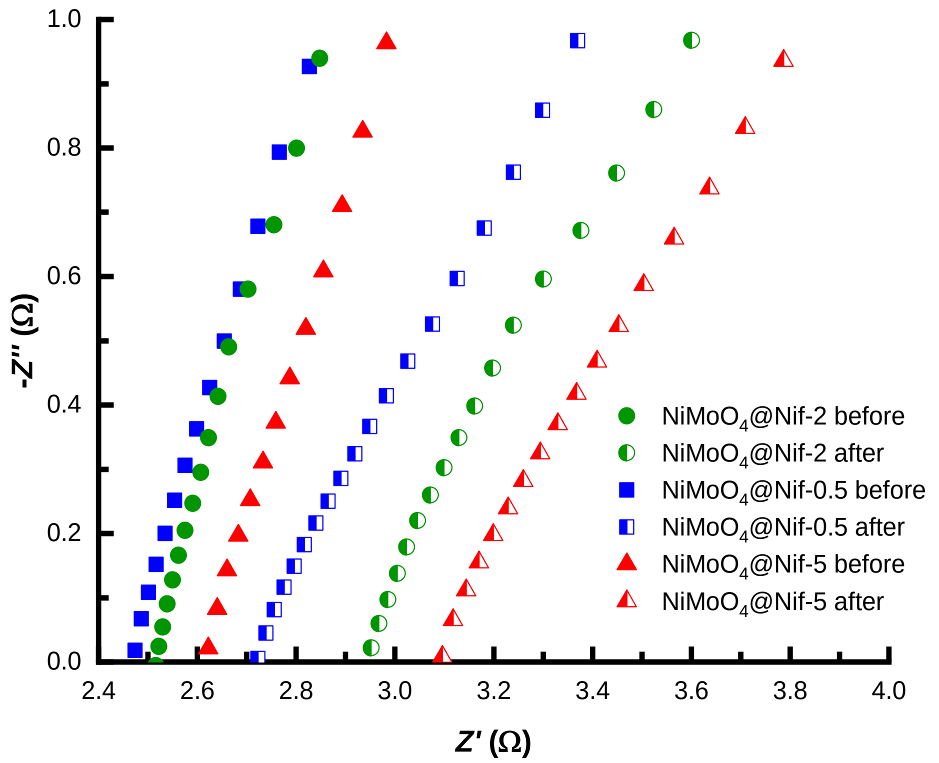

SI Figure 20: Magnification of the Nyquist plot in 1.0 M KOH on the ohmic resistance of the solution at 1.25 V *vs* RHE before (filled symbols) and after 500 cycles (half-filled symbols) for $NiMoO_4$@Nif-2 (green circles), $NiMoO_4$@Nif-0.5 (blue cubes) and $NiMoO_4$@Nif-5 (red triangles).

The Nyquist plot shows an increase of the ohmic drop between before and after 500 cycles. The resistance for $NiMoO_4$@Nif-2 from 2.52 Ω to 2.95 Ω, for $NiMoO_4$@Nif-0.5 increased from 2.47 Ω to 2.72 Ω and for $NiMoO_4$@Nif-5 from 2.62 Ω to 3.10 Ω.

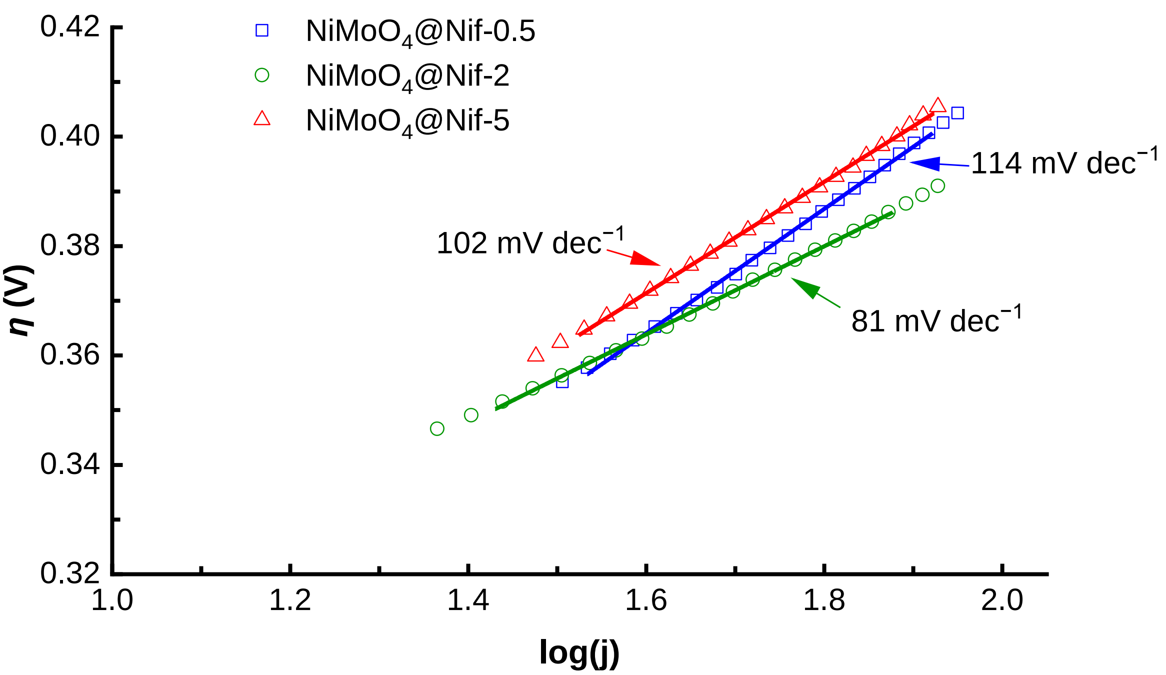

SI Figure 21: Tafel plot analysis of one of the very last cycles. The backwards scan of the 500th cycle of $NiMoO_4$@Nif-0.5 (blue hollow square) and $NiMoO_4$@Nif-5 (red hollow triangle) and the 499th cycle of $NiMoO_4$@Nif-2 (green hollow circle) was full *i*R compensated.

Tafel plot analysis was done for the backwards scan to reduce the influence of the Ni^{III}/Ni^{II} redox couple. The scan was manual fully iR compensated according to the solution resistance detected with EIS after 500 cycles.

CV with normalization to geometric surface area of nickel foam

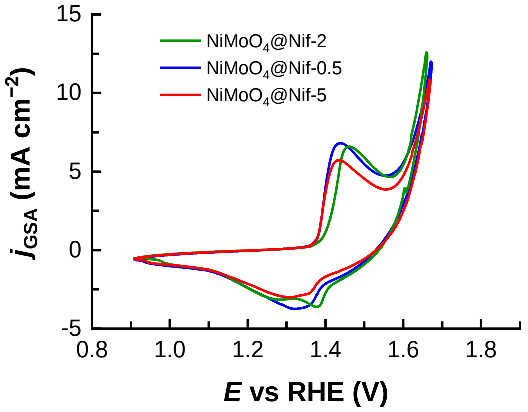

SI Figure 22: CV normalized to the geometric surface area of the nickel foam for better comparison of the performance of the catalyst. CV was taken with 10 mV s^{-1} in 1.0 M KOH with full manual *i*R compensation.

The 500th CV of each sample was full *i*R compensated and the current density normalized to the geometric surface area (GSA) of the blank nickel foam, which was provided by the supplier with 6.0 m^2 L^{-1}. The CV reveals an overpotential for 10 mA cm^{-2} of 410 – 430 mV. The 500th cycle was chosen, since the *i*R was directly obtained afterwards by EIS. One of the first cycle would have not experienced the activation step and a cycle in between would have an unknown *i*R.

SEM after 500 cycles in 1.0 M KOH

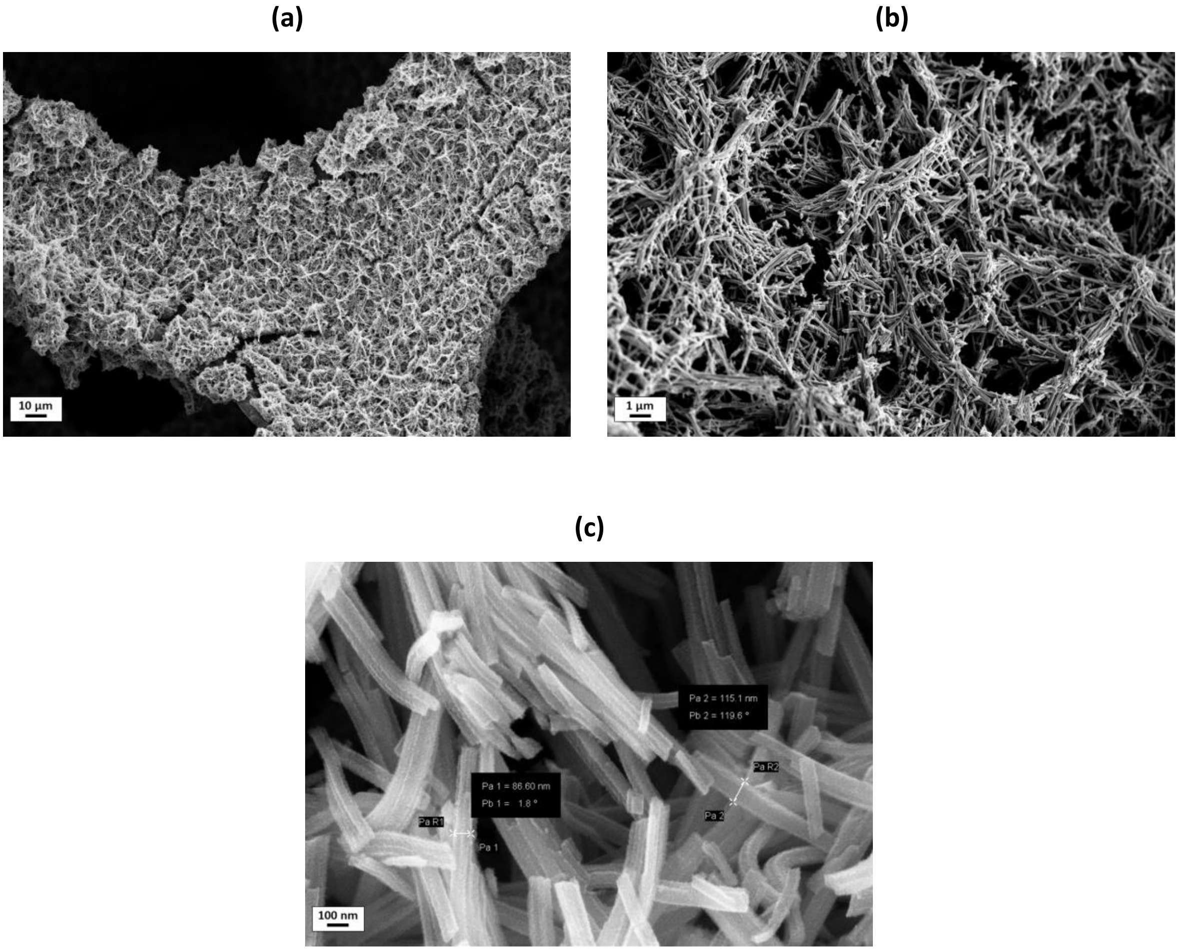

SI Figure 23: (a) – **(c)** SEM secondary electron images of $NiMoO_4$@Nif-2 after 500 cycles with different magnifications.

$NiMoO_4$@Nif-2 after 500 cycles showed slightly worn nanostructures. The nanorods seems to be broken and fused together, with a roughened surface. The macrostructure shows a higher porosity with spherical pores.

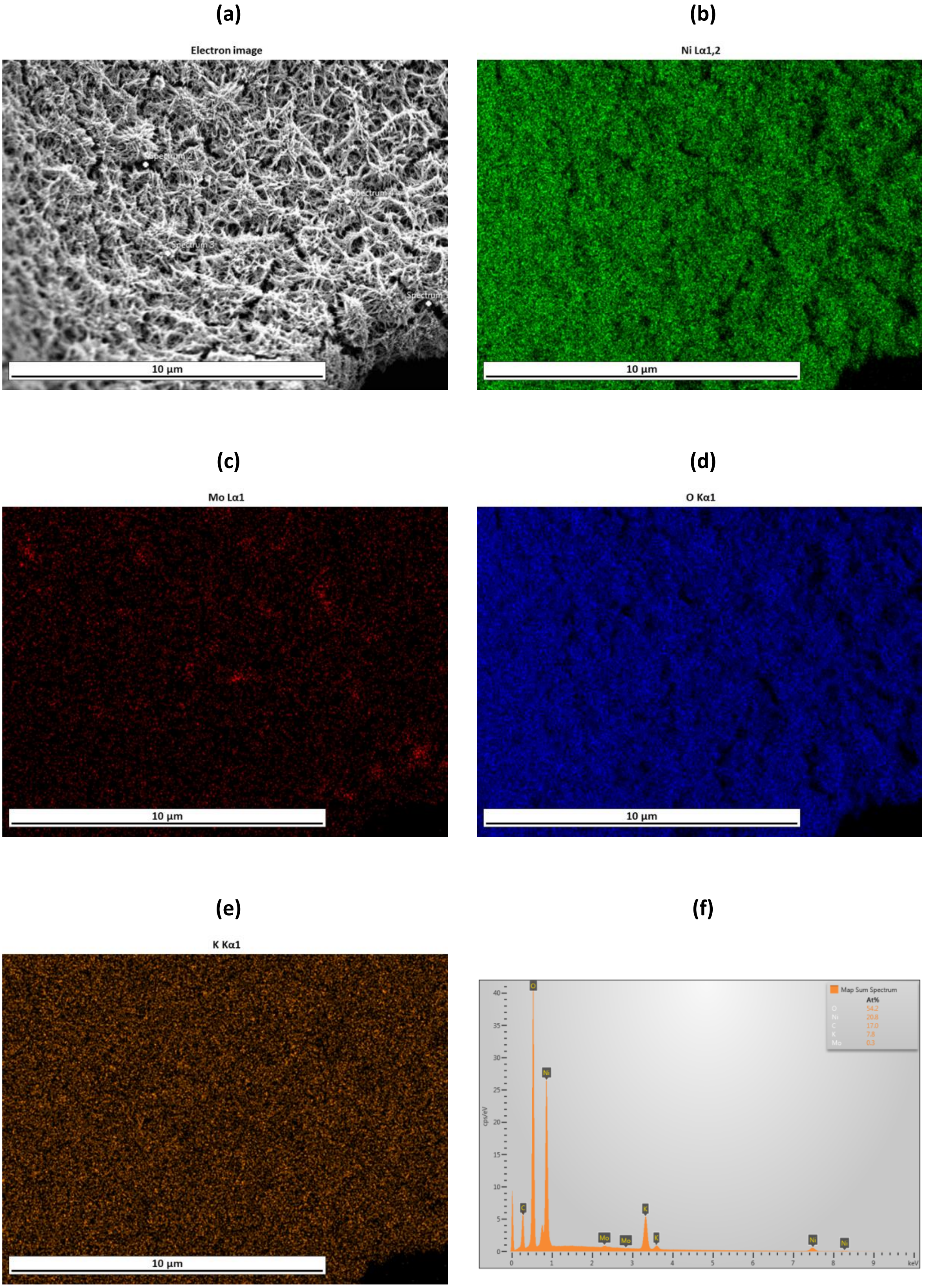

SI Figure 24: EDX analysis of $NiMoO_4$@Nif-2 after 500 cycles. **(a)** Secondary electron image of mapping area. **(b)** Nickel distribution. **(c)** Molybdenum distribution. **(d)** Oxygen distribution. **(e)** Potassium distribution. **(f)** EDX spectrum of the area.

EDX analysis of $NiMoO_4$@Nif-2 after 500 cycles revealed the removal of molybdenum from the material and the presence of potassium.

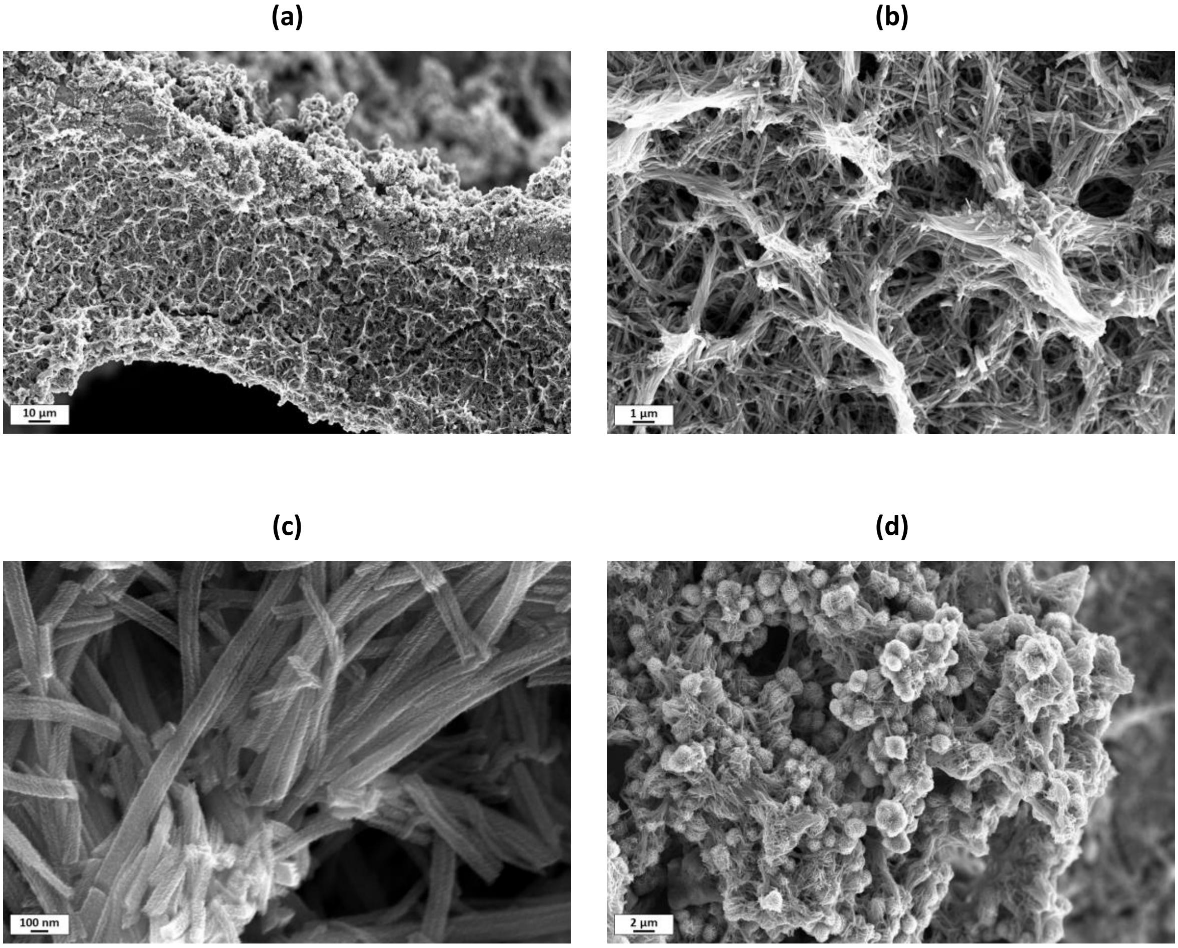

SI Figure 25: (a) – **(d)** SEM secondary electron images of $NiMoO_4$@Nif-0.5 after 500 cycles with different magnifications.

The nanostructure on $NiMoO_4$@Nif-0.5 after 500 cycles showed both, nanorods and nanoflowers. Big open pores were created and the nanorods seem to be fused together. Furthermore, the surface of those rods seems to be extremely roughened. Regions with a high amount of nanoflowers were also detected.

(a) **(b)**

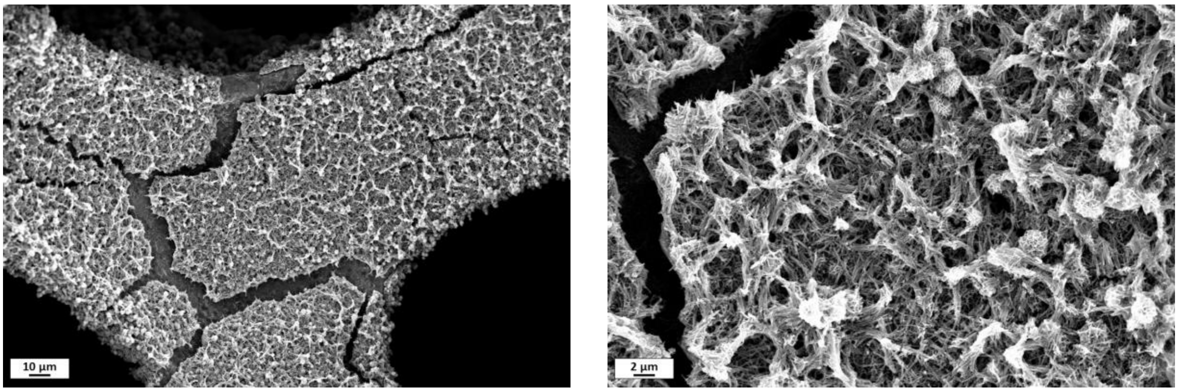

(c)

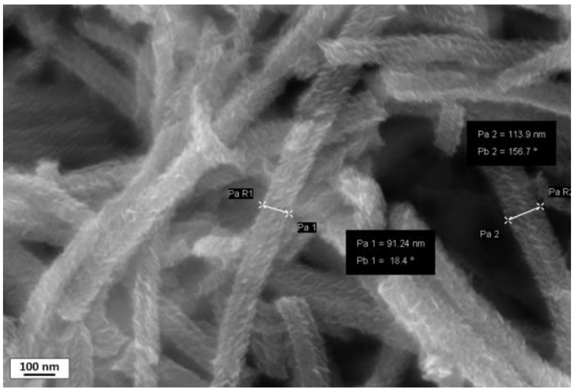

SI Figure 26: (a) – **(c)** SEM secondary electron images of $NiMoO_4$@Nif-5 after 500 cycles with different magnifications.

The SEM investigation of $NiMoO_4$@Nif-5 after 500 cycles confirmed what was seen for the other samples. A higher open porosity and roughened nanorods.

XPS after 500 cycles in 1.0 M KOH

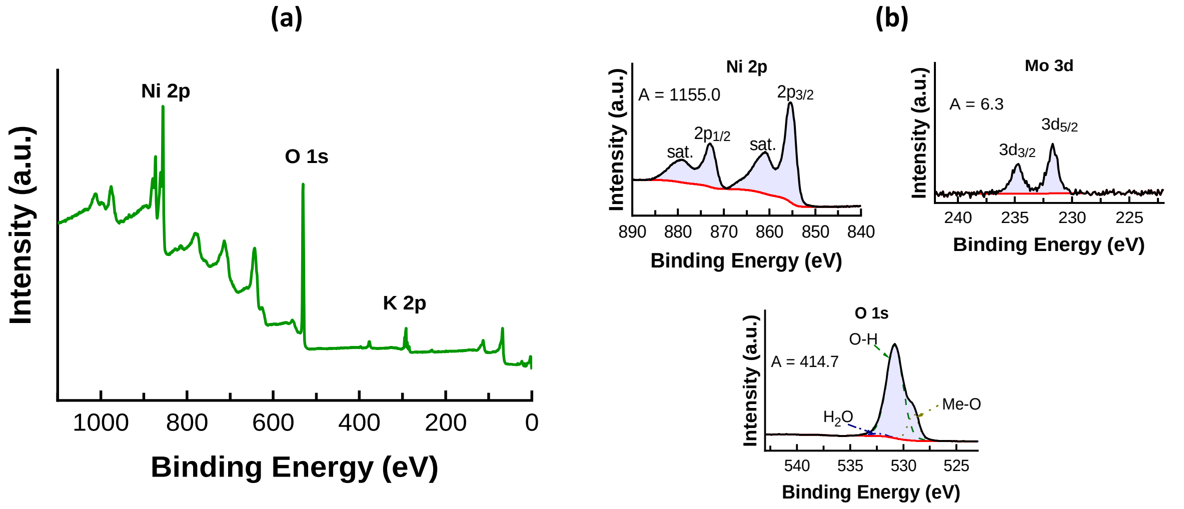

SI Figure 27: XPS spectra of $NiMoO_4$@Nif-2 after 500 cycles in 1.0 M KOH. **(a)** Survey spectrum. **(b)** High resolution elemental analysis of Ni 2p, Mo 3d and O 1s.

The XPS spectra of $NiMoO_4$@Nif-2 are discussed in the main publication.

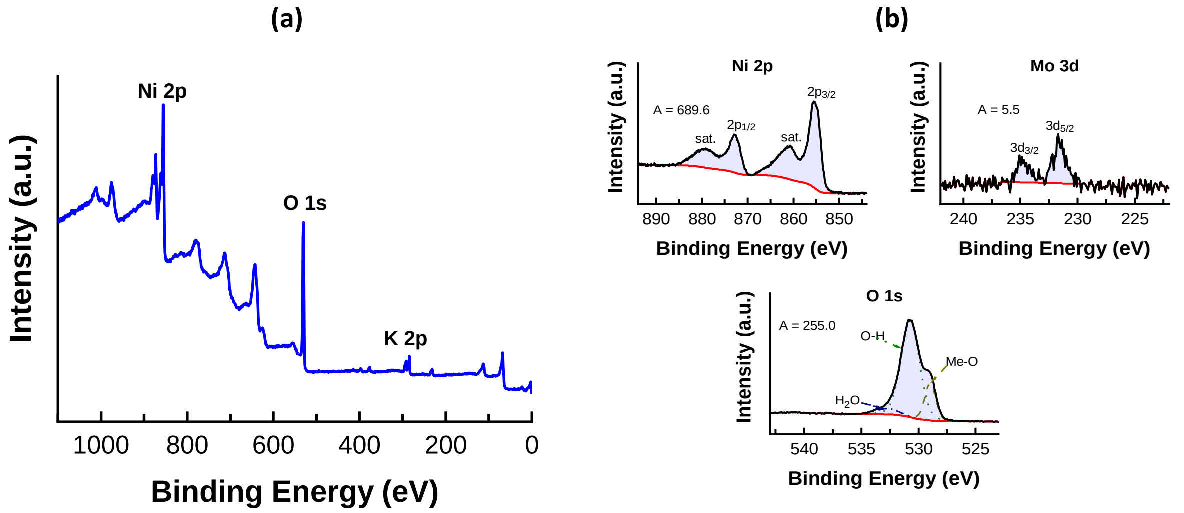

SI Figure 28: XPS analysis of $NiMoO_4$@Nif-0.5 after electrochemical testing in 1.0 M KOH. **(a)** Survey spectrum. **(b)** High resolution elemental analysis of Ni 2p, Mo 3d and O 1s.

$NiMoO_4$@Nif-0.5 after electrochemical treatment confirms the presence of potassium as detected by EDX. Only a very low amount of molybdenum was detected. The nickel peaks for $2p_{3/2}$ and $2p_{1/2}$ were appear at 855.4 eV and 872.8 eV, respectively. The oxygen shows a majority of hydroxides with a decrease amount of metal oxide. Also, some water was detected. The corresponding peaks and their

contribution to the overall oxygen peak are 5.6 at.% water at 532.6 eV, 17.9 at.% metal oxide at 529.0 eV and 76.5 at.% hydroxide at 530.7 eV.

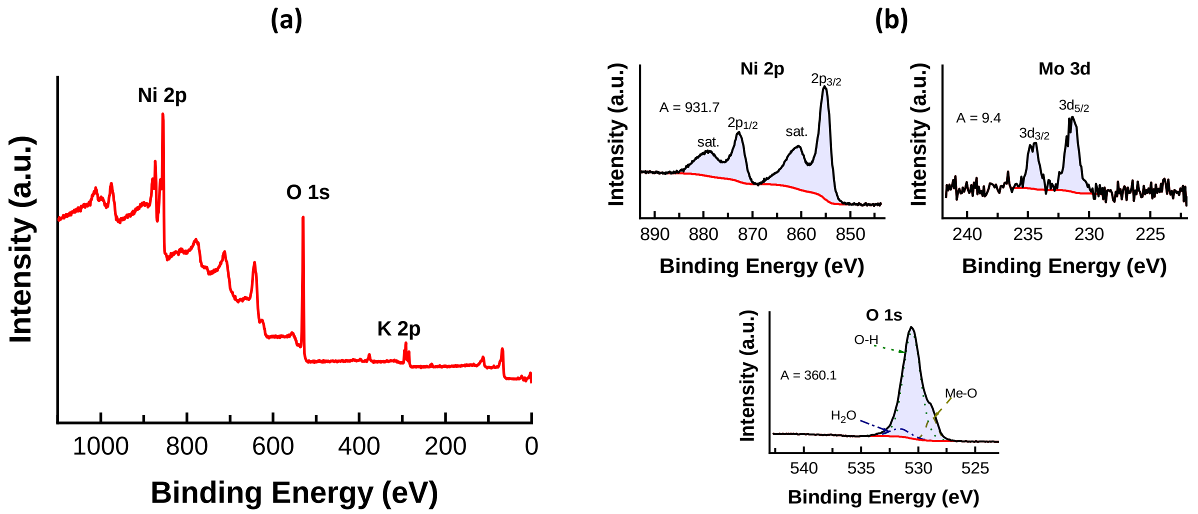

SI Figure 29: XPS analysis of $NiMoO_4$@Nif-5 after electrochemistry in 1.0 M KOH. **(a)** Survey spectrum. **(b)** High resolution elemental analysis of Ni 2p, Mo 3d and O 1s.

As for the other samples, $NiMoO_4$@Nif-5 after electrochemistry shows the presence of potassium and a drastic decrease of molybdenum in the material. The nickel $2p_{3/2}$ and $2p_{1/2}$ peak appeared at 855.2 eV and 872.9 eV. The oxygen peak could be divided in three fittings. One for water at 531.6 eV, a second for hydroxide at 530.5 eV and one for lattice metal oxide at 528.8 eV. Based on those fittings the composition was calculated as 7.4 at.% from H_2O, 11.8 at.% metal oxide and 80.8 at.% hydroxide. As for the others, the electrochemical process increased the amount of hydroxide drastically.

According to the work of Biesinger *et al.* [4], the presence of γ-NiOOH can be detected by a small peak in the Ni LMM Auger spectra at around 832.8 eV kinetic energy. As described in the time resolved *operando* Raman spectroscopy section, we identified γ-NiOOH as the active species. Since the XPS was not done directly after the electrochemical measurements, we believe γ-NiOOH has transformed over time in another nickel-(oxy)hydroxide and therefore we cannot detect the characteristic small peak for γ-NiOOH in our spectra.

SI Table 6: Calculation of elemental ratio of $NiMoO_4$@Nif after 500 cycles after excluding H_2O contribution.

	Element	Area	RSF	Corrected RSF	Area/RSF	at.%
$NiMoO_4$@Nif-2	Ni 2p	1155.0	3.702	4.05739	284.67	33.9
	Mo 3d	6.3	3.544	4.03071	1.56	0.2
	O 1s	406.0	0.733	0.733	553.88	65.9
$NiMoO_4$@Nif-0.5	Ni 2p	689.6	3.702	4.05739	169.96	34.0
	Mo 3d	5.5	3.544	4.03071	1.36	0.3
	O 1s	240.7	0.733	0.733	328.40	65.7
$NiMoO_4$@Nif-5	Ni 2p	931.7	3.702	4.05739	229.63	33.4
	Mo 3d	9.4	3.544	4.03071	2.33	0.3
	O 1s	333.5	0.733	0.733	454.91	66.2

Selective Mo etched $NiMoO_4$@Nif-5

(a) (b)

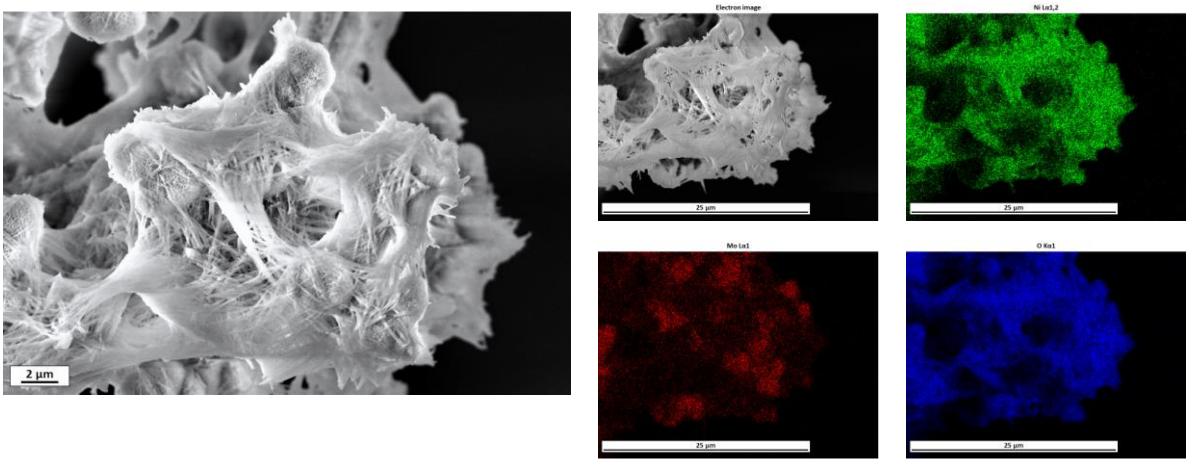

SI Figure 30: Selective molybdenum etched $NiMoO_4$@Nif-5. **(a)** SEM secondary electron image of the area, which is investigated by EDX mapping. **(b)** EDX mapping of etched sample with the electron image, nickel, molybdenum and oxygen.

After the selective molybdenum etching of $NiMoO_4$@Nif-5 in 1.0 M KOH without applying bias, the Raman spectrum only showed the presence of one molybdate structure. EDX mapping confirmed molybdenum only being present in the flower like structure. The concentration of nickel and oxygen seemed unchanged.

Time resolved *in situ* and *operando* Raman spectroscopy

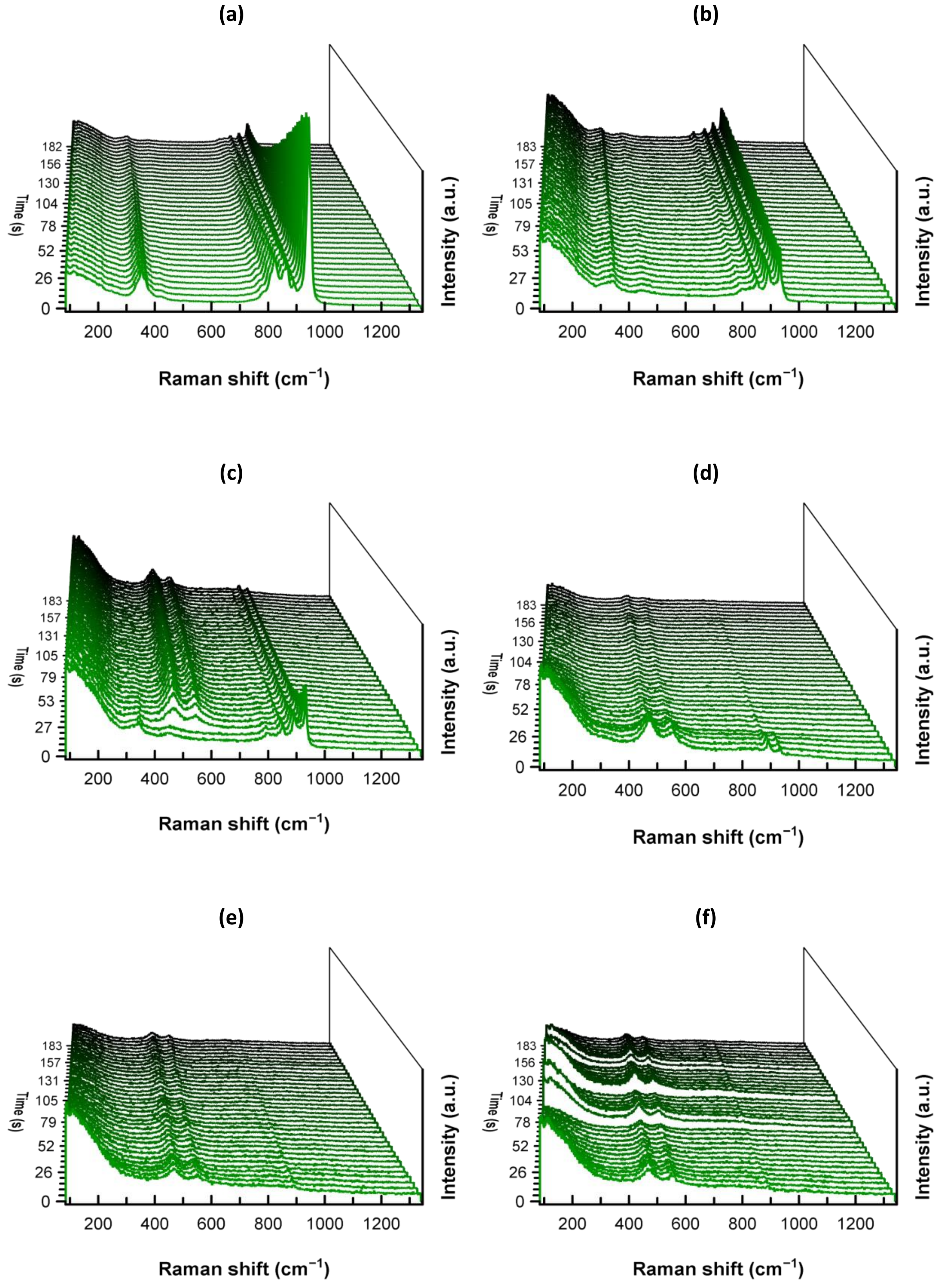

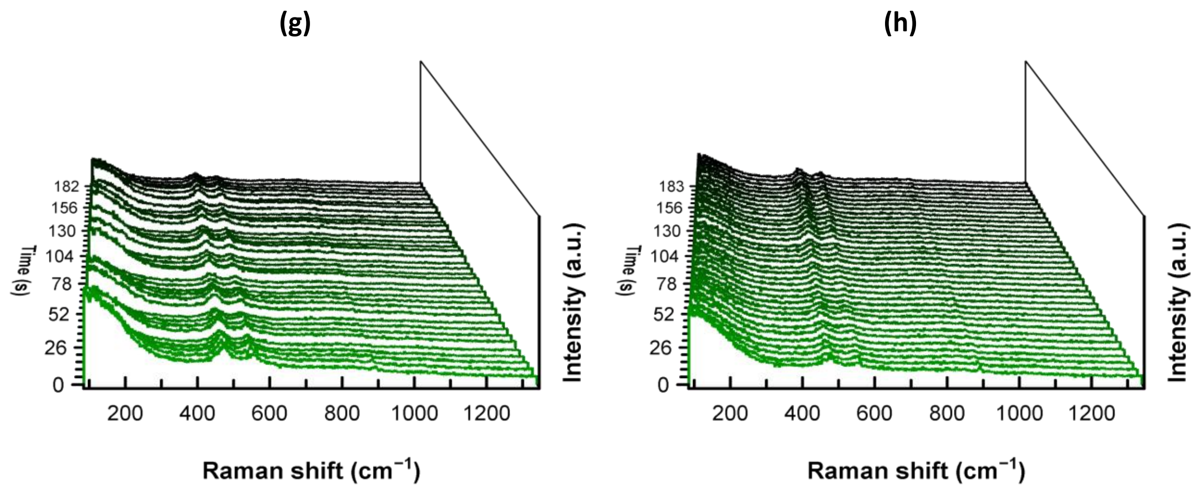

SI Figure 31: Time resolved Raman spectroscopy of $NiMoO_4$@Nif-2 in 1.0 M KOH over 3 minutes each. Laser intensity of 50 % with a 10 × magnification lens and acquiring time of 5 s was used for each spectrum. **(a)** *In situ* before CPE. **(b)** *In situ* during CPE at 0.95 V *vs* RHE. **(c)** *In situ* during CPE at 1.4 V *vs* RHE. **(d)** *In situ* during CPE at 1.5 V *vs* RHE. **(e)** *Operando* during CPE at 1.6 V *vs* RHE. **(f)** *Operando* during CPE at 1.7 V *vs* RHE. **(g)** *Operando* during CPE at 1.8 V *vs* RHE. **(h)** *In situ* after CPE.

Time resolved *in situ* and *operando* Raman spectroscopy of $NiMoO_4$@Nif-2 shows the instantaneous removal of the rod-$NiMoO_4$ without applying any bias. The vibrations of flower-$NiMoO_4$ vanishes during the oxidation of Ni^{III}/Ni^{II} with the simultaneous formation of γ-NiOOH.

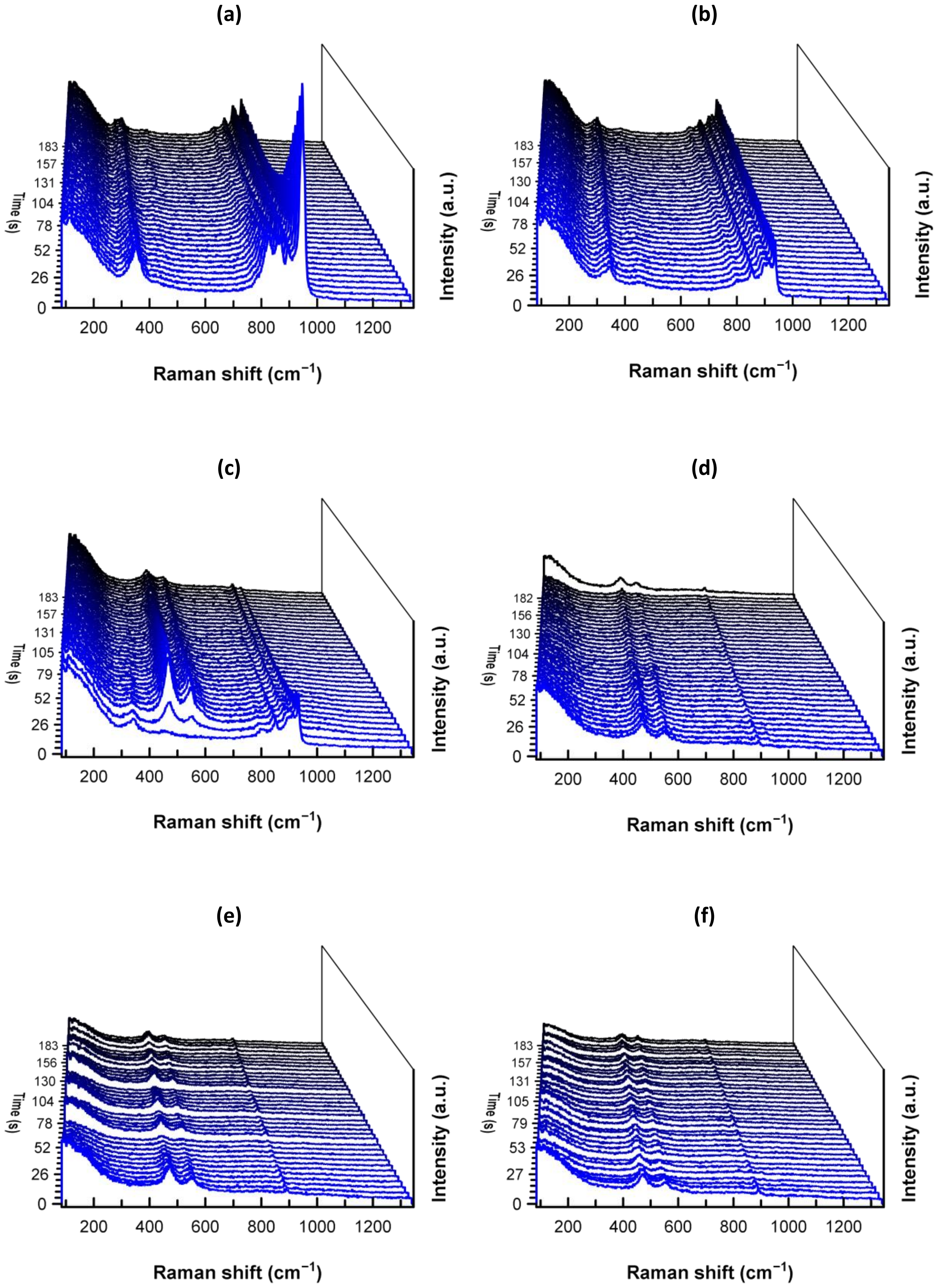
(a)
(b)
(c)
(d)
(e)
(f)
Time (s)
Intensity (a.u.)
Raman shift (cm−1)

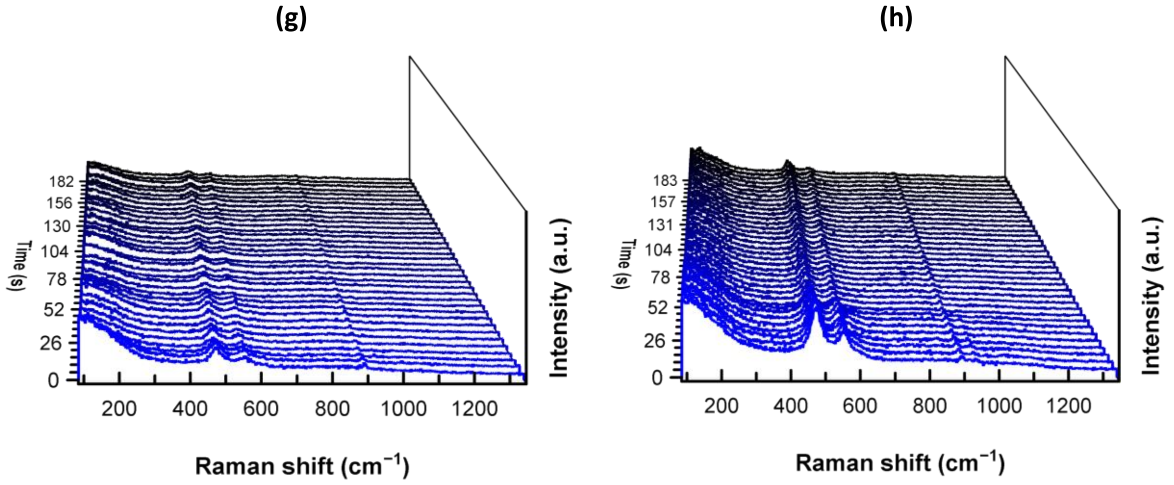

SI Figure 32: Time resolved Raman spectroscopy of $NiMoO_4$@Nif-0.5 in 1.0 M KOH over 3 minutes each. Laser intensity of 50 % with a 10 × magnification lens and acquiring time of 5 s was used for each spectrum. **(a)** *In situ* before CPE. **(b)** *In situ* during CPE at 0.95 V *vs* RHE. **(c)** *In situ* during CPE at 1.4 V *vs* RHE. **(d)** *In situ* during CPE at 1.5 V *vs* RHE. **(e)** *Operando* during CPE at 1.6 V *vs* RHE. **(f)** *Operando* during CPE at 1.7 V *vs* RHE. **(g)** *Operando* during CPE at 1.8 V *vs* RHE. **(h)** *In situ* after CPE.

Time resolved *in situ* and *operando* Raman spectroscopy of $NiMoO_4$@Nif-0.5 shows the same trend as $NiMoO_4$@Nif-2. The instantaneous removal of the rod-$NiMoO_4$ during *in situ* and the disappearing of the flower-$NiMoO_4$ vibrations during the oxidation of Ni^{III}/Ni^{II} with the simultaneous formation of γ-NiOOH.

(a) (b)

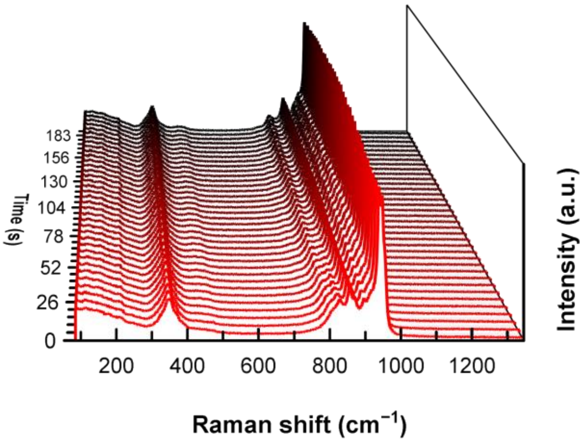

183
156
130
104
78
52
26
0
Time (s)
200 400 600 800 1000 1200
Raman shift (cm⁻¹)
Intensity (a.u.)

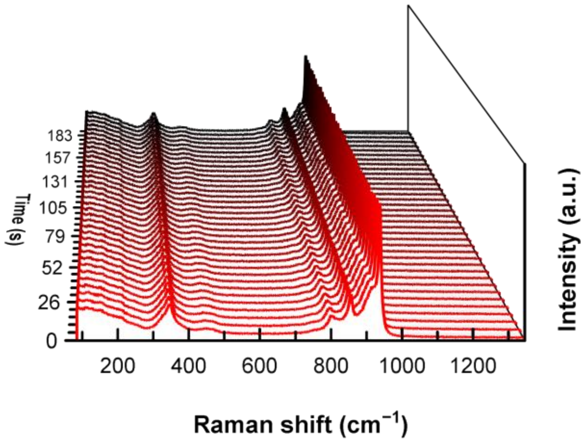

183
157
131
105
79
52
26
0
Time (s)
200 400 600 800 1000 1200
Raman shift (cm⁻¹)
Intensity (a.u.)

(c) (d)

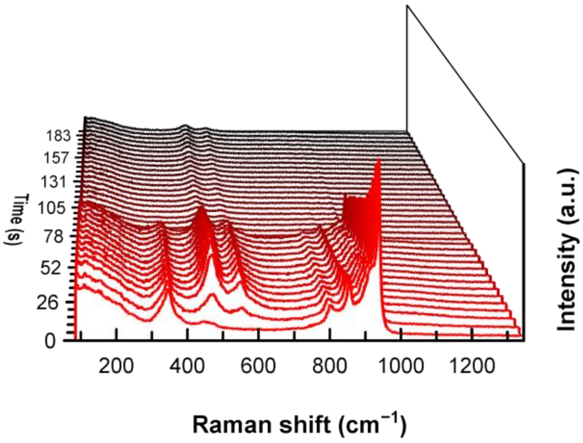

183
157
131
105
78
52
26
0
Time (s)
200 400 600 800 1000 1200
Raman shift (cm⁻¹)
Intensity (a.u.)

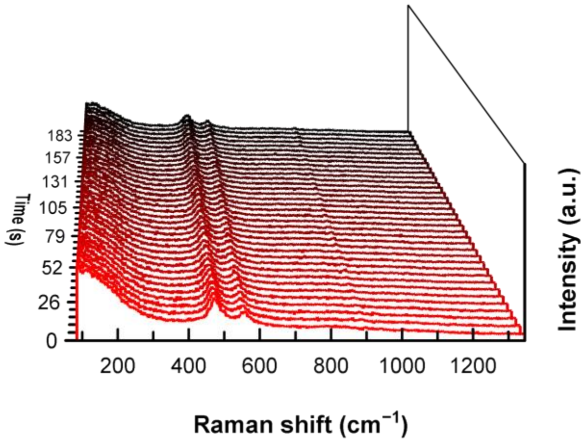

183
157
131
105
79
52
26
0
Time (s)
200 400 600 800 1000 1200
Raman shift (cm⁻¹)
Intensity (a.u.)

(e) (f)

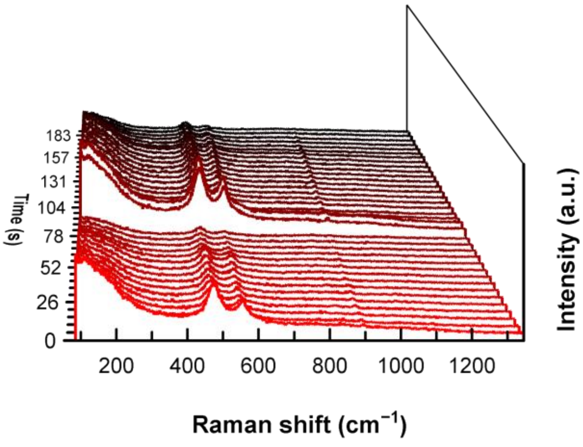

183
157
131
104
78
52
26
0
Time (s)
200 400 600 800 1000 1200
Raman shift (cm⁻¹)
Intensity (a.u.)

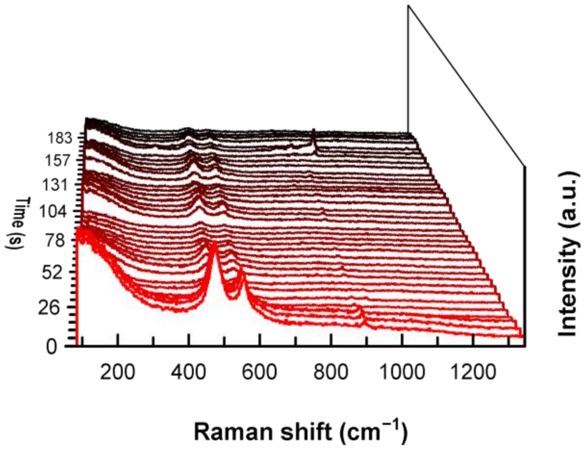

183
157
131
104
78
52
26
0
Time (s)
200 400 600 800 1000 1200
Raman shift (cm⁻¹)
Intensity (a.u.)

(g) (h)

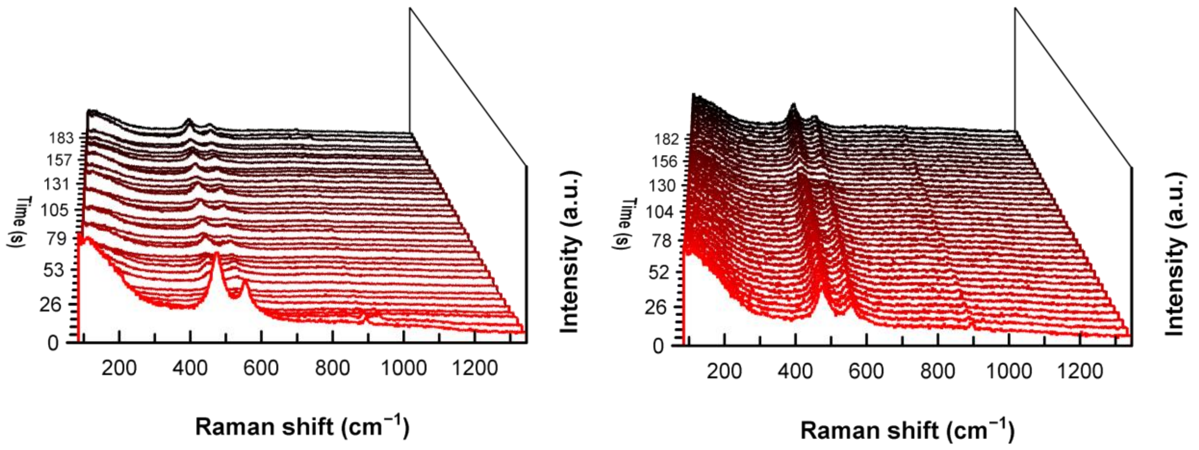

SI Figure 33: Time resolved Raman spectroscopy of $NiMoO_4$@Nif-5 in 1.0 M KOH over 3 minutes each. Laser intensity of 50 % with a 10 × magnification lens and acquiring time of 5 s was used for each spectrum. **(a)** *In situ* before CPE. **(b)** *In situ* during CPE at 0.95 V *vs* RHE. **(c)** *In situ* during CPE at 1.4 V *vs* RHE. **(d)** *In situ* during CPE at 1.5 V *vs* RHE. **(e)** *Operando* during CPE at 1.6 V *vs* RHE. **(f)** *Operando* during CPE at 1.7 V *vs* RHE. **(g)** *Operando* during CPE at 1.8 V *vs* RHE. **(h)** *In situ* after CPE.

Time resolved *in situ* and *operando* Raman spectroscopy of $NiMoO_4$@Nif-5 confirms what was already seen for the other samples.

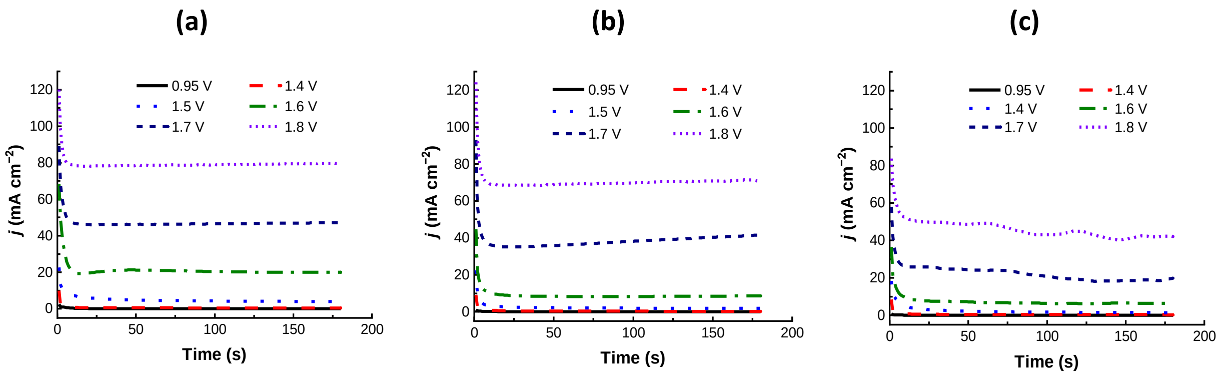

SI Figure 34: CPE in 1.0 M KOH for *in situ* and *operando* Raman acquisition. All potentials are reported *versus* RHE. **(a)** $NiMoO_4$@Nif-2. **(b)** $NiMoO_4$@Nif-0.5. **(c)** $NiMoO_4$@Nif-5.

SI Figure 34 shows the CPE used in *in situ* and *operando* Raman spectroscopy in **SI Figure 32** - **SI Figure 33**.

ICP-OES after CPE

The loss of molybdenum (Mo) in the catalyst into the electrolyte was quantified with inductively coupled plasma optical emission spectrometry (ICP-OES).

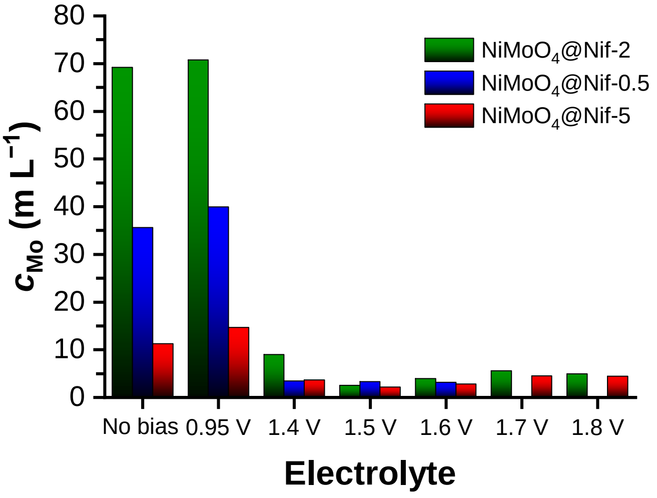

SI Figure 35: Concentration of molybdenum in electrolyte of CPE (potentials *vs* RHE), detected by ICP-OES.

SI Table 7: Molybdenum concentration and RSD value detected by ICP-OES.

Electrolyte	$NiMoO_4$@Nif-2		$NiMoO_4$@Nif-0.5		$NiMoO_4$@Nif-5	
	c_{Mo} (mg L^{-1})	RSD (%)	c_{Mo} (mg L^{-1})	RSD (%)	c_{Mo} (mg L^{-1})	RSD (%)
In situ	69.219	0.8	35.604	3	11.203	0.8
0.95 V	70.76	1.6	39.931	3	14.635	1.5
1.4 V	8.923	0.4	3.423	0.6	3.592	0.3
1.5 V	2.481	1.9	3.271	0.4	2.135	0.9
1.6 V	3.942	1.2	3.134	0.6	2.775	0.4
1.7 V	5.527	0.8	5.002	0.2	4.441	0.2
1.8 V	4.899	0.8	4.371	1.9	4.439	0.3

EDX after CPE

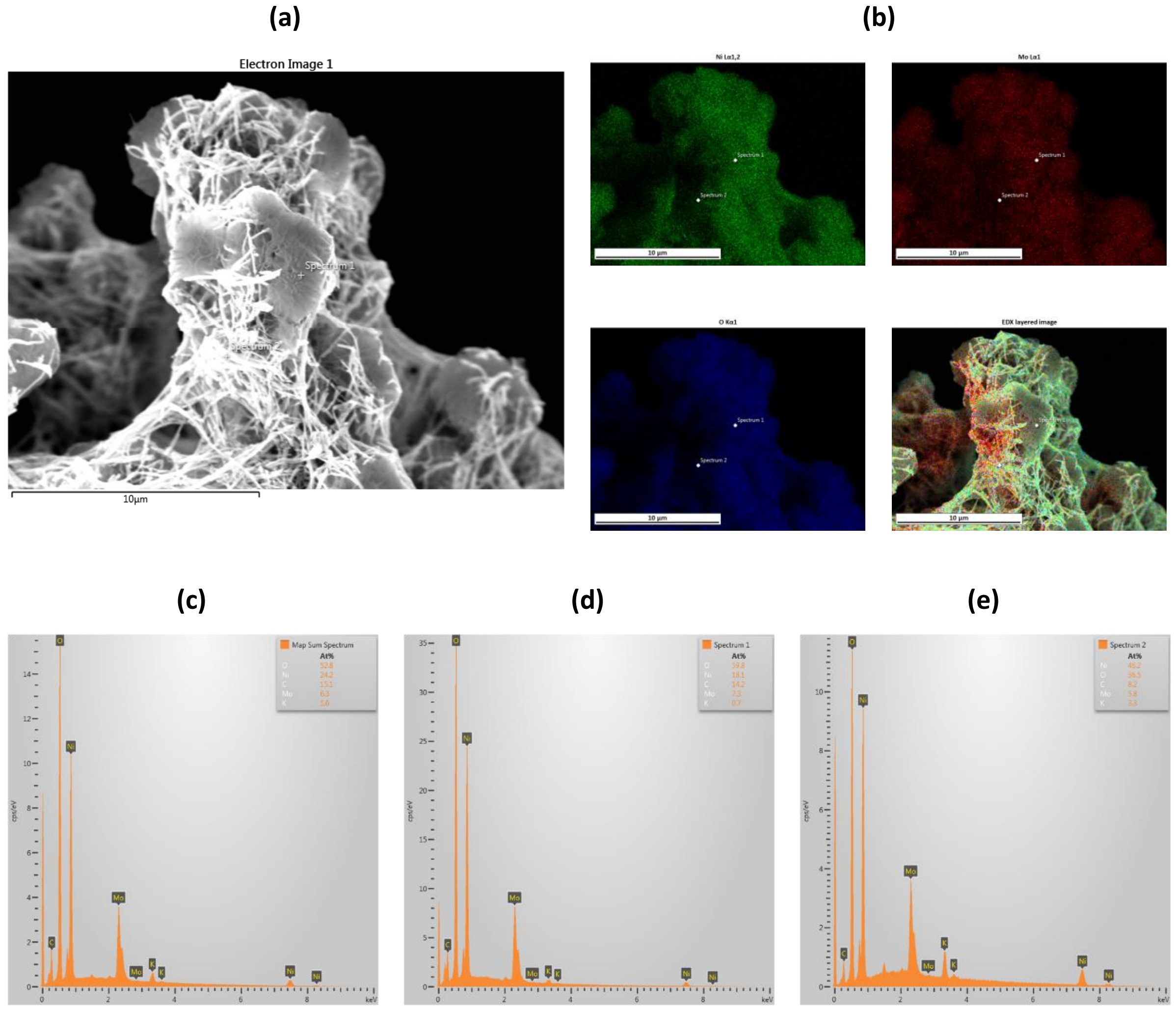

SI Figure 36: EDX investigation of $NiMoO_4$@Nif-5 after CPE. **(a)** Secondary electron image of mapping area with the spots of the two point ID spectra. **(b)** Elemental mapping of nickel, molybdenum and oxygen and its overlay. **(c)** – **(e)** EDX spectra of mapping area and the two point ID spots.

EDX after controlled potential electrolysis (CPE) shows a decreased concentration of molybdenum, however still present and detectable.

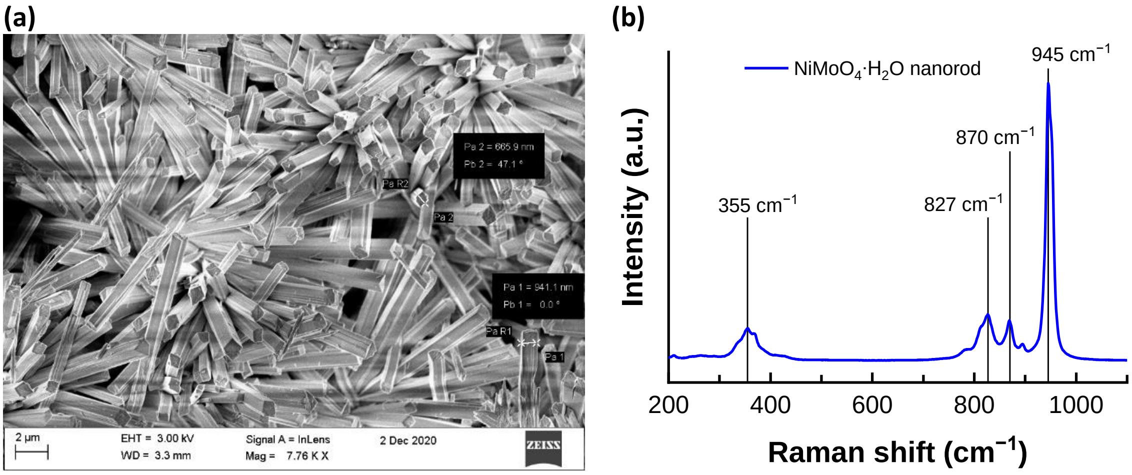

SI Figure 37: $NiMoO_4 \cdot H_2O$ nanorods synthesized with ammonium heptamolybdate tetrahydrate instead of sodium molybdate dihydrate. **(a)** Secondary electron (SE) image showing thick nanorods. **(b)** *Ex situ* Raman spectrum showing corresponding rod-$NiMoO_4$ vibrations.

As control experiment, rod-$NiMoO_4$ was synthesized with using ammonium heptamolybdate tetrahydrate as molybdate source, to suppress the formation of flower-$NiMoO_4$. The slight shift of wavenumbers for the same vibrations (356.5 cm^{-1}, 827.9 cm^{-1}, 872.0 cm^{-1}, 948 cm^{-1} when using $Na_2MoO_4 \cdot H_2O$) can be explained by a possible overlay of spectra from different molybdate species, when using the $Na_2MoO_4 \cdot H_2O$.

Bibliography

1. Wang, J.; Li, L.; Meng, L.; Wang, L.; Liu, Y.; Li, W.; Sun, W.; Li, G. Morphology Engineering of Nickel Molybdate Hydrate Nanoarray for Electrocatalytic Overall Water Splitting: From Nanorod to Nanosheet. *RSC Adv.* **2018**, 8, 35131-35138.

2. Zhang, J.; Wang, T.; Liu, P.; Liao, Z.; Liu, S.; Zhuang, X.; Chen, M.; Zschech, E.; Feng, X. Efficient Hydrogen Production on $MoNi_4$ Electrocatalysts with Fast Water Dissociation Kinetics. *Nat. Commun.* **2017**, 8, 1-8.

3. Rountree, E. S.; McCarthy, B. D.; Eisenhart, T. T.; Dempsey, J. L. Evaluation of Homogeneous Electrocatalysts by Cyclic Voltammetry. *Inorg. Chem.* **2014**, 53, 9983–10002.

4. Biesinger, M. C.; Lau, L. W. M.; Gerson, A. R.; Smart, R. S. C. The Role of the Auger Parameter in XPS Studies of Nickel Metal, Halides and Oxides. *Phys. Chem. Chem. Phys.* **2012**, 14, 2434–2442.
